# Evolutionary and structural pathways of antibody maturation reveal principles for engineering potent SARS-CoV-2 neutralizers

**DOI:** 10.1016/j.yjsbx.2026.100161

**Published:** 2026-09-18

**Authors:** Muhammad Waqas Nasir, Qiyun Liang, Jing Wang, Yan Sun, Xiangyu Zhang, Rishen Liang, Jun He, Umer Rashid, Jiabing Wu, Yong Gao

**Affiliations:** a The First Affiliated Hospital of USTC, Division of Life Sciences and Medicine, University of Science and Technology of China, Hefei, Anhui 230001, China; b Department of Chemistry, University of Science and Technology of China, Hefei, Anhui 230001, China; c Anhui Provincial Center for Disease Control and Prevention, Hefei 230601, China; d Hefei Infectious Disease Hospital, Hefei 230001, China; e Department of Chemistry, COMSATS University, Islamabad, Abbottabad Campus, 22060 Abbottabad, Pakistan

**Keywords:** SARS-CoV-2, Affinity maturation, IGHV4–34 lineage, CDRH3 somatic hypermutation, SYP cap, Antibody engineering

## Abstract

The continual emergence of SARS-CoV-2 variants underscores the need to elucidate antibody maturation and apply rational design principles to optimize therapeutics. This study focused on vaccine-elicited 9C lineage, encoded by IGHV4–34, as a model for affinity maturation and structural refinement. Sequence analyses revealed strong framework conservation alongside focused diversification within CDRH3, where the invariant RWFD anchor and a recurrent SYTV block were consistently associated with potent neutralization. Phylogenetic reconstruction traced a trajectory from weakly neutralizing precursors, such as 9A91, to ultrapotent clones, like 9C2, showing nearly a 3000-fold improvement in IC₅₀ values. Computational modeling, molecular dynamics, and docking analyses demonstrated that potency arises from progressive stabilization of the CDRH3 apex, with the SYT motif at VH106–108 serving as a critical structural driver with improved IC_50_ = 5.52 μg/mL. Residues at positions 98, 102, and 105 further fine-tuned stability and binding geometry, while rigid versus flexible apex conformations revealed convergent solutions for high-affinity binding. Engineered variants of 9A91 recapitulated these principles, with SYT installation and flanking substituents improving stability, energetics, and antigen engagement. ELISA validation confirmed up to 64% enhanced binding and neutralization with IC_50_ = 0.76 μg/mL compared with the germline precursor. Collectively, these findings define a hierarchical mechanism of antibody maturation, where conserved anchoring motifs, apex stabilization, and secondary refinements synergize to achieve potency, establishing a framework for rational antibody design against SARS-CoV-2 and emerging viral threats.

## Graphical abstract

**Figure:**
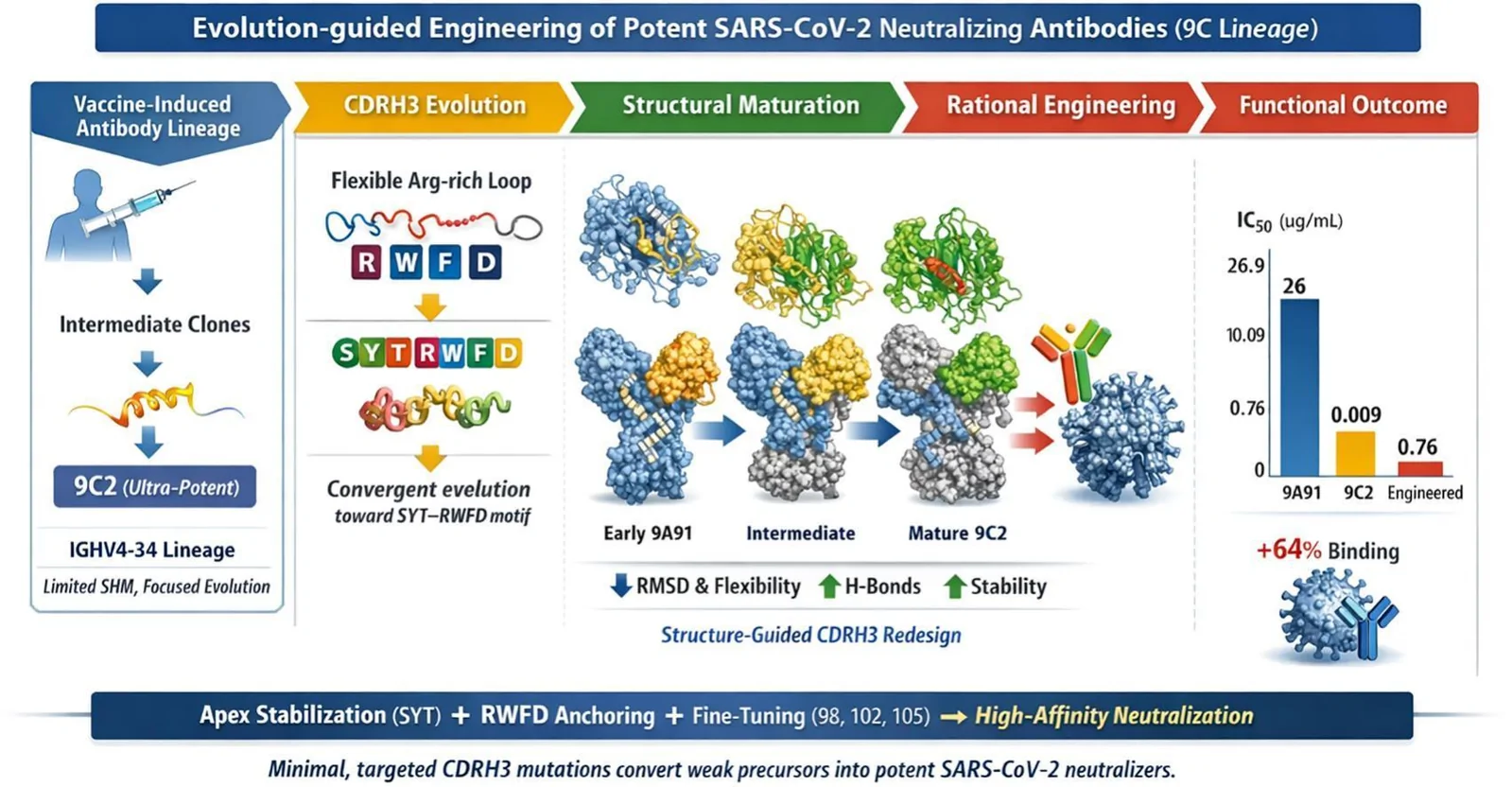

## 1 Introduction

The COVID-19 pandemic has highlighted the urgency of understanding how protective antibodies arise, mature, and persist under different immunological conditions (Mohan et al., 2023). Monoclonal antibodies (mAbs) have been central to therapeutic interventions and serve as powerful models for examining the principles of B-cell evolution following infection or vaccination(Pantaleo et al., 2022). In contrast to natural infection, which commonly induces extensive somatic hypermutation (SHM) and broad neutralization, vaccine-induced responses typically evolve through more constrained yet reproducible evolutionary pathways (Yu et al., 2023). Defining the genetic and structural determinants of these pathways is essential for rational immunogen design and the engineering of durable therapeutics active against evolving SARS-CoV-2 lineages (Naqvi et al., 2020).

Somatic hypermutation (SHM) introduces mutations into immunoglobulin variable regions during germinal-center reactions and, through positive selection, derives affinity maturation (Eid et al., 2017). Tracing SHM patterns within clonal families enables reconstruction of evolutionary trajectories, identification of minimal mutational requirements for enhanced binding, which probed by rational site-directed mutagenesis of CDRH3 (triple substitution installing an SYT cap at VH106–108; single-site edits at positions 98, 102, 103, and 105 such as G102A and Y105R; combinatorial variants; and reciprocal residue swaps between high- and intermediate-potency clones) and recognition of conserved motifs that serve as anchors for antigen engagement (Tas et al., 2016), (Gupta et al., 2017). Vaccine-derived antibodies are particularly informative in this respect, as their maturation occurs under controlled antigenic exposure and population-level reproducibility, allowing generalizable insights for vaccine development (Choy et al., n.d.).

Previous studies have identified several “public” antibody lineages, such as IGHV3–30, IGHV3–33, IGHV3–53, IGHV1–58, and IGHV1–24, that recur across individuals following SARS-CoV-2 infection. By contrast, vaccine-induced responses may follow distinct evolutionary trajectories, shaped by the nature of controlled antigen exposure (Kuwata et al., 2024). Notably, the IGHV4–34 lineage has recently been recognized as a novel vaccine-elicited neutralizing antibody family with a strong correlation to immune protection. Antibodies within this lineage, such as the potent 9C2 clone, illustrate how convergent structural motifs like the SYTV-RWFD configuration in CDRH3 enable high-affinity binding and efficient viral neutralization with relatively limited mutational burden (Wang et al., 2024a).

Integration of clonal genetics with structure-guided computational approaches provides further insight into the molecular basis of 9C2 activity. Homology modeling, docking, and molecular dynamics simulations clarify the stabilizing role of the SYTV-RWFD configuration at the antigen-binding interface, quantify interaction energetics, and suggest potential mutations that may enhance affinity or breadth. Somatic hypermutation along with selective pressure progressively enhances antibody binding and specificity during immune evolution (Zuo et al., 2025).

However, the structural mechanisms involved in driving these enhancements are not fully understood. This study analyzes these pathways, yields mechanistic insights into protective immunity and informs strategies for developing next-generation antibodies with improved potency and breadth. By defining the minimal sequence and structural determinants of vaccine-induced neutralizing activity, this study establishes principles that can guide rational vaccine design and computer-aided antibody engineering (Guarra and Colombo, 2023). Accordingly, a comprehensive investigation of the evolution, structure, and functional properties of vaccine-induced antibodies is critical, as such studies not only advance our understanding of protective immunity guide the development of next-generation vaccines and therapeutic antibodies that can counter SARS-CoV-2 variants and emerging viral threats (Dormitzer et al., 2008). In future, discovering the molecular mechanisms underlying antibody maturation is essential for the progression of vaccine and therapeutic development against other viral diseases as well.

## 2 Materials and methods

### 2.1 Antibody source and sequence dataset

The vaccine-derived monoclonal antibody 9C2 and its clonotypes (9A91, 9B65, 9C111, 9C11, 9C154, 9C30, and 9C101) were isolated from peripheral B cells of individuals immunized with the inactivated SARS-CoV-2 vaccine (Sinopharm)(Wang et al., 2024b). In this investigation, we utilized all vaccinee #09 reported light and heavy chain sequences for recombinant production in HEK293F cells. All sequences corresponded to productive, in-frame rearrangements free from stop codons or frameshifts. No additional human volunteers were recruited. All experimental procedures utilized recombinant antibodies and recombinant SARS-CoV-2 receptor-binding domains (RBD) proteins specific to the Wuhan-Hu-1.

### 2.2 Recombinant RBDs expression and purification

DNA fragments encoding the receptor-binding domains (RBD) of the SARS-CoV-2 variants, specifically spike residues 319–541, were codon-optimized for expression in human cells and synthesized by SnapGene. His tags were appended to the terminal ends of the RBD gene fragments. The fragments were subsequently incorporated into pTT5 expression vectors. The primer sequences were modified to include SAI I and Bam HI restriction enzyme sites, respectively. Sangon Biotech (Shanghai) Co., Ltd. produced the primer sequences used to amplify the SARS-CoV-2 RBD target segment. Using particular primers, the RBD fragment was amplified by polymerase chain reaction and subsequently cultured for plasmid extraction. HEK293F cells underwent transfection using the designed plasmids and were cultured for a duration of six days. The purification of the products was conducted through Ni-NTA columns (New dragon biotechnology, Jiangxi), and the resulting samples were confirmed through SDS-PAGE analysis. Tengchuan Jin of China's University of Science and Technology kindly donated the pTT5 vector and the HEK293F cell line.

### 2.3 V(*D*)J assignment and junctional analysis

VH sequences were analyzed using IgBlast (NCBI) (https://www.ncbi.nlm.nih.gov/igblast/) with the IMGT germline database (https://www.imgt.org/) as reference. V, D, and J gene assignments were recorded with their corresponding percentage identities. Junctional analysis was performed to extract complementarity-determining region H3 (CDRH3) sequences, which were then assessed for motif recurrence, amino acid length variation, and junctional diversity. Annotations were cross-validated using IMGT/HighV-QUEST (https://www.imgt.org/highvquest/) (Ye et al., 2013).

### 2.4 Sequence alignment, conservation profiling, and clonal tracing

VH protein sequences were globally aligned using Clustal Omega (https://www.ebi.ac.uk/jdispatcher/msa/clustalo) to assess residue-level conservation across the IGHV4–34 clonal family. Shannon entropy was calculated for each alignment column, and consensus fractions were used to classify conserved residues. Sequence logo plots were generated using WebLogo v3.7 (http://weblogo.threeplusone.com/) where positions with consensus fraction ≥0.80 were defined as highly conserved and those with entropy >1.5 bits as variable. Clonal tracing employed CLUSTAL W (https://www.ebi.ac.uk/Tools/msa/clustalw2/) to derive a majority-rule consensus sequence as a proxy for the unmutated common ancestor (UCA) (Crooks et al., 2004; Sievers and Higgins, 2018). Divergence between clonotypes and the UCA was quantified by computing pairwise distances (p-distances) across 125 aligned amino acid positions. Radial phylogenetic diagrams were constructed to visualize lineage topology and relative divergence within the IGHV4–34 family.

### 2.5 Structural modeling and docking

The Fv fragment of the 9C family was modeled using Alphafold2, and the Galaxy web refiner was used to refine the structure. Structural models were energy-minimized and stereo-chemically validated using MolProbity (https://molprobity.biochem.duke.edu/index.php) via clash scores and Ramachandran statistics. Docking studies with the SARS-CoV-2 receptor-binding domain (RBD) (PDB ID:9UTF) were performed using ClusPro (https://cluspro.bu.edu/) and HDOCK (http://hdock.phys.hust.edu.cn/) servers, treating the Fvs as receptor and RBD as ligand. Docked complexes were ranked based on buried surface area, hydrogen-bonding networks, and interface energetics(Yan et al., 2020; Kozakov et al., 2017; Davis et al., 2007).

### 2.6 Validation of docking protocol

Docking validation was conducted to determine the validity of the computational modeling approach. The experimentally determined XG83 Fab-RBD complex (PDB ID: 9UTF) used as a benchmark for evaluating docking precision. The receptor binding domain (RBD) was re-docked into the XG83 antibody with identical docking parameters, and the anticipated conformation was placed in juxtaposition with the crystallographic structure. The resultant RMSD among docked versus experimental configurations was below 2.0 Å, validating the precision of the docking procedure. Interface investigations utilizing PDBePISA validated constant hydrogen bonding in addition shape compatibility with the original complex. Additionally, 100 ns of molecular dynamics simulations were conducted on the highest-ranked docked complex to assess its durability, hydrogen bond retention, and binding free energy, thereby confirming the reliability of the docking predictions.

### 2.7 Molecular dynamics simulations

Molecular dynamics (MD) simulations were conducted using GROMACS 2024 (http://www.gromacs.org/) with the CHARMM36 force field (https://charmm.org/). Fab-RBD complexes were placed in a triclinic simulation box with 10 Å padding, solvated with explicit TIP3P water molecules, and neutralized with counter-ions. Ionic concentration was adjusted to 0.15 M NaCl to mimic physiological conditions. Energy minimization was performed using the steepest descent algorithm to eliminate steric clashes (Abraham et al., 2015; Bjelkmar et al., 2010). The system was equilibrated in two stages: (i) constant volume and temperature (NVT) equilibration for 100 ps at 300 K using the V-rescale thermostat, and (ii) constant pressure and temperature (NPT) equilibration for 100 ps at 1 bar with the Parrinello-Rahman barostat. During equilibration, protein heavy atoms were restrained to allow solvent relaxation. A 100 ns production simulation was conducted under periodic boundary conditions, with trajectory frames saved every 2 ps. Analyses included root-mean-square deviation (RMSD) to evaluate global system stability, root-mean-square fluctuation (RMSF) to characterize residue flexibility, radius of gyration (Rg) to assess compactness, and solvent-accessible surface area (SASA) to measure conformational changes in antigen-binding loops. Hydrogen bonding persistence across the Fab-RBD interface was quantified(Lemkul, 2024).

Binding energetics were estimated using energy trajectory files of Gromacs. Free energy decomposition identified residues with dominant energetic contributions to binding. Principal component analysis (PCA) was applied to MD trajectories to capture major conformational states. These dynamic and energetic analyses complement static docking predictions.

### 2.8 Motif and Interface analysis

Heavy-chain CDR3 regions of all clonotypes were examined for conserved sequence features. Structural superimposition confirmed the presence of the SYTV cap upstream of the invariant RWFD motif. Residue-level interactions were mapped using PyMOL (https://pymol.org/) and MOE (2016,0802), to highlight hydrogen bonds, hydrophobic contacts, and electrostatic complementarity at the Fv-RBD interface. These analyses clarify the structural contribution of conserved motifs to antigen binding.

### 2.9 Mutated antibody plasmid construction, expression, and purification

The SARS-CoV-2-specific mutated antibody expression vectors were produced using the VRC01 heavy and light chain expression plasmids(Xie et al., 2021) that contains the IgG1 constant sections of human from the NIH, along with the CMV promoter and Kana resistance. These vectors were generated from the human immunodeficiency virus (HIV) neutralizing antibodies. The heavy chain variable region sequences and the light chain variable region sequences were cloned independently into the antibodies' expression vectors discussed before to generate an antibody. Xba1、Sal1 restriction enzyme sites were used. Variable regions coding genes for paired light and heavy chains of vaccine# 09 mutated antibodies were synthesized (Shanghai, Sangon Biotech) and subsequently cloned into heavy and light chain expression vectors and subsequently cultured for plasmid extraction. Plasmid was extracted by the EndoFree maxi plasmid kit (Tiangen). The corresponding antibodies were produced through transient transfection of HEK293F cells. Equal amounts of heavy and light chain expression plasmids (50 μg for 50 ml culture) were *co*-transfected into HEK293F cells using polyethyleneimine (PEI, Polyscience) at a density of 2 × 10^6^ cells/ml. After five days, cell culture supernatants were collected and purified using a Protein A column (GE Healthcare), followed by centrifugation with 10 kDa MWCO membrane centrifugal filter units (Millipore). The purified antibodies were quantified using a Nanodrop spectrophotometer and BSA kit (Biotech), dispensed in aliquots of 100 g each, and stored at −80 °C until use.

### 2.10 Enzyme-linked immunosorbent assay (ELISA)

A 200 ng/well coating of stabilized soluble SARS-CoV-2 spike protein RBD in 1× PBS was applied to ELISA plates (BIOFIL) and left at 4 °C for 16 h. The coated wells were then incubated at 37 °C for 2–3 h with 200 μL of blocking buffer, which is PBS with 5% BSA. Afterwards, 300 μL of washing buffer, which is PBS with 0.1% Tween 20, was used to wash the wells three times. The SARS-COV-2 spike protein-coated wells were incubated in a series of dilutions of the wild types and mutated antibodies for 1 h at 37 °C. PBS was utilized as a blank control. Following three washes with washing buffer, the wells were incubated for 1 h at 37 °C with a 1:250 dilution of goat anti-human IgG secondary antibody. After adding the TMB (Beyotime) substrate and letting it react in the dark for 15 min, we added the Stop Solution to stop the colour development. An Elisa Analyzer (Agilent) was used to detect absorbance at 450 nm.

### 2.11 Pseudo-virus neutralization

This section details the procedures used to conduct a neutralization assay with SARS-CoV-2 pseudoviruse D614G in order to determine whether 9A91 mutants were effective in blocking D614G variant entry. 96-well plates were seeded with 10,000 cells/well of HEK293T/hACE2 cells in culture media twenty-four hours prior to the test. After being five-fold serially diluted in culture medium, the testing antibodies were incubated with 50 μL of pseudoviruse at 37 °C for one hour. The initial dilution for antibodies dilution was 1 mg/mL or 10 μg/mL. After that, HEK293T/hACE2 cells were treated with the antibodies and virus mixture and left to incubate at 37 °C with 5% CO2 for an additional 48 h. Lastly, the Britelite plus Reporter Gene Assay System (PerkinElmer) was used to evaluate the luciferase activity of HEK293T/hACE2 cells infected with Pseudovirus. Utilizing fitting nonlinear regression curves in GraphPad Prism 10.4.1, we were able to calculate the highest antibody dilution that resulted in 50% pseudoviruse neutralization (NTIC50).

### 2.12 SDS-PAGE analysis of purified antibodies

Following recombinant expression in HEK293F cells and purification using Protein A affinity chromatography, the parental and engineered antibodies of the 9C lineage were evaluated by sodium dodecyl sulfate-polyacrylamide gel electrophoresis (SDS-PAGE) to assess protein integrity and the presence of the expected immunoglobulin chains. Purified antibody samples were subjected to SDS-PAGE under conditions allowing separation of the heavy- and light-chain components, and a molecular-weight protein marker covering 25–180 kDa was included for size estimation. Following electrophoretic separation, the gels were visualized and examined for the characteristic antibody heavy-chain and light-chain bands. SDS-PAGE profiles were obtained for 9A91 and its engineered derivatives, as well as the 9C-lineage antibodies and their corresponding mutants. The resulting gel images were used as quality-control evidence for the recombinant antibody preparations prior to subsequent binding and functional assays.

### 2.13 Statistical analysis

By comparing the RLU values with the negative control wells, the OD450 was determined using GraphPad Prism 10 (GraphPad Software), transformed the data by using a standard function (transform X values using X = logX), and produced the non-linear curve as the dilution at which the RLU values were reduced. GraphPad Prism 10 was used to examine the statistical significance of the difference in OD450. A non-linear regression analysis was conducted to examine the relationship between anti-SARS-CoV-2 antibodies and RBDs, determining the binding affinity by plotting the curves.

## 3 Results

### 3.1 Conserved germline usage with junctional diversity

Analysis of the 9C2 clonal family revealed a strikingly conserved origin from the germline heavy chain variable gene IGHV4–34*01, with all eight clonotypes sharing between 92.3% and 93.9% identity. This uniformity highlights a repertoire strongly biased toward a single germline segment, suggesting shared selective pressure favoring this framework. In contrast, greater heterogeneity was observed in the D gene assignments. While most clonotypes, including 9A91, 9B65, 9C111, 9C11, 9C154, and 9C30, employed IGHD2–2 alleles, two clonotypes diverged: 9C101 aligned with IGHD4–11/40, and 9C2 with IGHD3–16*03. J gene usage was comparatively constrained, dominated by IGHJ5*02, with minor variation in 9C30, 9C101, and 9C2, which aligned to IGHJ5*01/02. All sequences were productive, in-frame, and free of stop codons or frameshifts, underscoring their positive selection as functional antigen receptors (Table S1).

Global sequence alignment highlighted that the CDR3 region accounted for the greatest degree of variability across the clonotypes (Fig. 1A). Most antibodies are encoded with 18-amino acid loops; however, 9A91 and 9B65 deviate with CDR3s composed of 19 and 15 residues, respectively. Despite this variability, a strong degree of convergence was observed in amino acid composition. In particular, the “SYTV-RWFD” motif was consistently preserved across potent neutralizers. This convergence is emphasized in the sequence logo, which shows a fully conserved RWFD block (positions 110–113), while diversity remains confined to a central stretch spanning positions 97–107 (Fig. 1B). These results underscore structural constraints that safeguard antigen recognition even amid sequence heterogeneity.

**Fig. 1:**
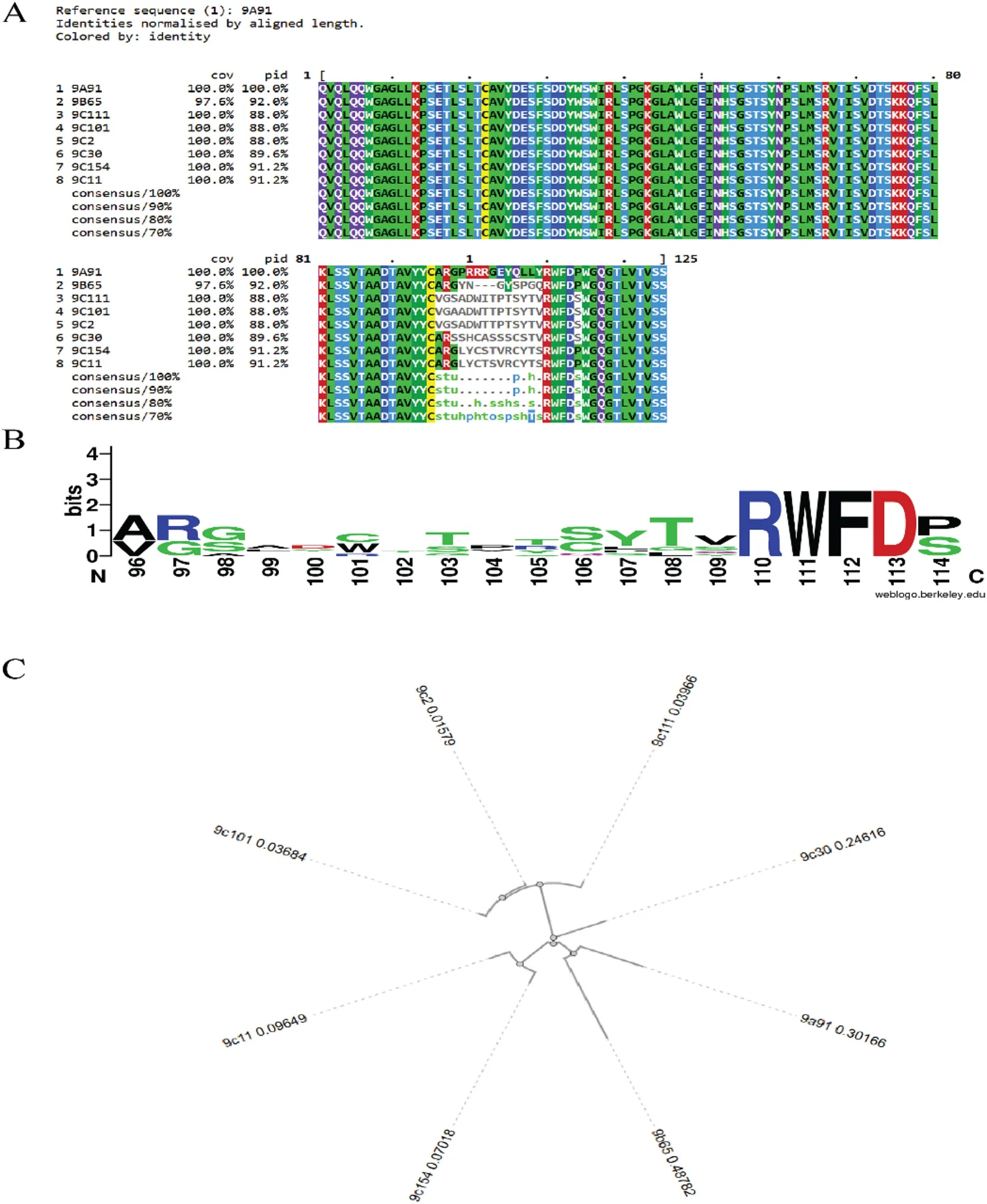
Sequence conservation and clonal tracing of the IGHV4–34 lineage. (A) Global alignment of eight clonotypes from the 9C2 lineage, showing high framework conservation and focal variability within the CDR3 loop. Most sequences encoded 18-residue CDR3s, while 9A91 and 9B65 deviated with 19 and 15 residues, respectively. Notable divergence occurs at positions 98 (Ala/Ser) and 102 (Ile/Thr), which distinguish late-branch antibodies 9C101 and 9C111 from the parental 9C2. Overall conservation remained high, with 88% of sites maintaining a consensus ≥0.80. (B) Sequence logo of the heavy-chain variable region (125 aa), illustrating positional information content. A fully conserved RWFD motif (positions 110–113) is preserved across potent neutralizers, while diversity is restricted to positions 97–107. (C) Radial lineage diagram constructed from global sequence alignment. The star-like topology, with short internal branches and pairwise distances ranging from 0.032 to 0.096, indicates rapid expansion from a recent unmutated common ancestor (UCA) and limited diversification.

Clonal tracing reinforced the picture of a lineage shaped by both conservation and limited diversification. Alignment of eight clonotypes revealed high overall conservation (88% of sites with consensus ≥0.80; mean entropy 0.191 bits). Radial lineage analysis produced a star-like topology with short internal branches and modest divergence, consistent with rapid expansion from a recent unmutated common ancestor (UCA). Pairwise distances ranged from 0.032 to 0.096, corresponding to only 4–12 substitutions across the alignment (Fig. 1C).

Taken together, these results define the 9C2 clonal family as a vaccine-elicited, IGHV4–34-restricted public antibody lineage, characterized by conserved frameworks, selective variability in the D gene, and strong convergence on the SYTV-RWFD motif within the CDR3 loop. The juxtaposition of rigidly conserved motifs with localized sequence flexibility suggests a maturation strategy that preserves antigen-binding determinants while allowing limited diversification to fine-tune potency.

### 3.2 Affinity maturation and functional diversification

To elucidate the evolutionary basis of antibody potency within the IGHV4–34 lineage, we reconstructed the clonal relationships among eight heavy-chain sequences isolated from vaccinee #9. Alignment of CDRH3 regions revealed strong conservation of the RWFD anchor motif (positions 110–113) and a recurring SYTV block immediately upstream, which was preferentially enriched in the most potent neutralizers. In contrast, low-potency precursors displayed greater sequence heterogeneity within this region. Notably, 9A91 retained a relatively arginine-enriched local sequence, whereas 9B65 did not exhibit a comparable Arg-rich pattern. Thus, the reduced potency of 9B65 should not be attributed to an arginine-rich CDRH3 sequence, indicating that additional sequence and structural features contribute to the functional differences among early-lineage antibodies. Phylogenetic reconstruction revealed a near star-like topology, showing that weakly neutralizing ancestors (9A91, IC₅₀ = 26.91 μg/mL; 9B65, IC₅₀ = 20.04 μg/mL) gave rise to progressively more potent descendants. The highest-affinity clones (9C2, IC₅₀ = 0.009 μg/mL; 9C11, IC₅₀ = 0.020 μg/mL; 9C111, IC₅₀ = 0.022 μg/mL) exhibited nearly 3000-fold improvements in neutralization relative to 9A91. This functional maturation was paralleled by sequence convergence: potent clones consistently adopted short polar motifs (SYTV-RWFD), whereas intermediate-potency members such as 9C30, 9C101, and 9C154 retained partial divergence (IC₅₀ = 0.120–0.160 μg/mL). Together, these data establish a hierarchy of functional tiers-low (9A91/9B65), intermediate (9C154/9C30/9C101), and high (9C2/9C11/9C111) emerging from the clonal network. The IC₅₀ values used in this analysis were obtained from previously published work (Wang et al., 2024b) (Fig. S1).

We profiled somatic hypermutation (SHM) across the D-region band immediately upstream of RWFD to identify where diversification was concentrated. Conservation tightened approaching the RWFD anchor, while the central residues (positions 98–105) tolerated frequent substitutions. Overall mutation frequency across this window was high (mean divergence 0.529), with the greatest variability in low-potency clones such as 9A91 (0.917) and 9C30 (0.833), while top-tier clones 9C2, 9C111, and 9C101 showed the least divergence (0.083). These findings suggest that affinity maturation favors controlled diversification within the central loop while stabilizing residues proximally to the RWFD anchor (Table 1).

**Table 1:** SHM in the D-region window per-sequence divergence.

Sequence	Mismatches	Sites compared	Per AA-distance
9A91	11	12	0.917
9C30	10	12	0.833
9B65	9	11	0.818
9C154	8	12	0.667
9C11	8	12	0.667
9C111	1	12	0.083
9C101	1	12	0.083
9C2	1	12	0.083

The sequence logo of heavy chains from vaccinee #9 revealed conservation across framework regions with diversification predominantly concentrated in the CDRH3 loop immediately upstream of the RWFD anchor. Early clones retained extended arginine-rich motifs, whereas later members showed progressive remodeling of this region. The most mature descendants converged on a compact SYTV block adjacent to RWFD, indicating strong selective pressure to preserve this structural motif. These observations confirmed that CDRH3 is the principal site of diversification, while framework and anchor motifs remain invariant to maintain structural integrity (Fig. S1).

### 3.3 Structural modeling and epitope prediction

To ensure accurate representation of variable regions, particularly the highly flexible CDRH3 loop, we benchmarked several modeling tools against the crystallographic structure of antibody XG83 (PDB ID:9UTF). Among Abodybuilder2, Molecular Operating Environment (MOE, 2016–0802), I-Tasser, Swiss-Model, and ColabFold (AlphaFold2), the latter provided the most reliable Fv models with CDRH3 RMSD values consistently below 1.5 Å (Table S2). Based on this performance, ColabFold was chosen to generate all subsequent models of the 9C lineage antibodies. The variable-domain (Fv) models of the eight lineage members were further refined to eliminate steric clashes and optimize stereochemistry. Refinement consistently reduced clash scores from elevated pre-refinement values (13.7–21.7) to 0.00, while MolProbity scores improved from ∼2.0 to <0.7, exceeding benchmarks for high-quality structural models. Ramachandran *Z*-scores clustered near zero, and all the residues are in favored regions, confirming plausible backbone geometries across both frameworks and CDRs (Table S3). These results demonstrate that the refined models meet rigorous structural standards and are suitable for downstream docking and epitope mapping.

Comparative analysis across the lineage showed that early clones (e.g., 9A91, 9B65, 9C30) retained open, solvent-exposed apices, while intermediate members (9C154, 9C101) adopted partially compact loops. Late high-potency clones (9C111, 9C11, 9C2) converged on a compact, turn-stabilized SYTV-RWFD motif, which repeatedly furnished the dominant paratope patch in Wuhan RBD docking runs.

This shift in apex geometry provides a structural explanation for the improved functional performance of mature lineage members. Superposed Fv models illustrate this trajectory clearly: frameworks remain stable, while CDRH3 evolves from short and kinked (9A91, 9B65) to elongated and turn-stabilized (9C111, 9C11, 9C2). The directional remodeling of CDRH3 explains how late-stage clones achieve enhanced neutralization by consolidating a hydrogen-bond-competent, geometrically focused apex immediately upstream of RWFD (Fig. S2).

### 3.4 Molecular docking study and interaction analysis

Docking simulations performed with ClusPro (Table 2) and HDOCK (Table S4) provided a comparative view of how antibodies from the 9C lineage engage the SARS-CoV-2 Wuhan RBD. Despite inherent differences in the docking engines, both methods converged on a consistent organizing principle: installation of the SYT cap (VH residues S106-Y107-T108) at the CDRH3 tip, immediately N-terminal to the invariant RWFD anchor, is the defining determinant of high-affinity binding.

**Table 2:** Molecular Docking Study and Interaction Analysis (Cluspro).

Antibody Name	Docking Score ClusPro	Antibody regions ClusPro	Antigen Regions Cluspro
9A91	−251.7	Heavy chain (A): GLU50, ASN52, SER56, THR57, SER58, MET64, ARG101, GLU104, TYR105, GLN106, LEU108, ARG110 Light Chain (B): TYR33, ASP93, ARG95	CHAIN A: THR345, ARG346, ALA348, SER349, ASN354, LYS356, LEU440, ASP441, LYS443, VAL444, GLY445, GLY446, ASN449
9B65	−312.0	Heavy chain (A): ASP26, GLU27, ASP31, LYS76, ARG97, TYR99, TYR102, GLN106, TRP108 Light Chain (B): ARG56, SER58, GLY59, ASP62	ARG346, TYR351, ALA352, ASN354, ARG355, ARG357, SER359, LYS443, ASN449, ARG456, GLU464, ARG465, ALE467, SER468, THR469, HIS518
9C111	−315.6	Heavy Chain (A): SER30, ASP31, ASP32, TYR33, GLU50, HIS53, SER56, TYR59, MET64, SER65, VAL67, THR105, SER106, TYR107, THR108, ARG110 Light Chain (B): TYR33, ARG95, SER98	LYS378, TYR380, ASP405, GLU406, ARG408, ALA411, GLY413, THR415, LYS417, ASP427, ASN459, ALA474, SER474, SER476, ASN486, TYR488, GLN492, GLY501, VAL502, GLY503, TYR504
9C154	−325.2	Heavy Chain (A): PHE29, SER30, ASP31, TYR33, HIS53, SER58, PRO61, SER102, VAL104, TYR107, ARG110 Light Chain (B): TYR93, ARG95, SER96, SER98	LYS417, LYS4457, ASN459, TYR472, ALA474, SER476, PHE485, ASN486, TYR488, GLN492, TYR504
9C30	−350.1	Heavy Chain (A): TYR33, SER54, GLY55, SER56, THR57, VAL71, ALA102, SER104, THR108, VAL109, ARG110 Light Chain (B): TYR33, TYR93, ARG95	GLY445, GLY446, TYR448, ASN449, THR477, CYS479, ASN480, GLY481, VAL482, GLU483, GLY484, PHE485, PHE489, PHE491, GLN492, SER493
9C101	−363.0	Heavy Chain (A): TYR33, THR57, SER58, TYR59, MET64, SER65, VAL67, THR68, LYS81, SER83, ASP100, SER106, TYR107, THR108, ARG110 Light Chain (B): TYR33, ARG95	LUS378, TYR380, ARG408, PRO412, GLN414, THR415, TYR421, LYS424, ASP427, ASP428, LYS457, ASN459, TYR472, ALA474, GLY475, SER476, ASN486
9C11	−359.5	Heavy Chain (A): SER28, ASP31, TYR33, GLU50, THR57, SER58, LYS76, CYS101, THR102, SER103, CYS106, ARG110 Light Chain (B): ALA31, TYR33, ASP34, TYR93, ARG95, LEU97, SER98	THR415, LYS417, TYR421, PHE455, ASN459, ALA474, SER476, ASN486, TYR488, TYR504
9C2	−370.1	Heavy chain (A): ASP26, GLU27, SER28, ASP31, ASP32, SER98, ASP100, THR102, SER106, TYR107, THR108, ASP113 Light Chain (B): SER58	ARG346, LYS443, VAL444, ASN447, TYR448, ASN449, GLU483, CYC487, PHE489, SER493

Across the lineage, the VH CDRH2 seat (S56-T57-S58) was recovered in nearly all docking poses, highlighting its role as a conserved anchoring feature. However, this seat alone did not discriminate potency, as even weak binders such as 9A91 and 9B65 relied on these residues without gaining strong docking scores. The decisive difference was the presence and consolidation of the SYT cap. Both docking platforms repeatedly identified S106, Y107, and T108 as core contact residues in potent clones, particularly in 9C2, 9C11, and 9C111 (Fig. S4). These residues form a compact hydrogen-bonding and π-stacking cluster that locks the CDRH3 apex into the RBD ridge, producing the most favorable docking energies.

Residue-level contact maps elucidate these distinctions. The CDRH2 seat (e.g., 9A91: S56-T57-S58) is retained across lineage members, but does not, on its own, distinguish strong binders. Instead, late-stage antibodies achieved enhanced binding by consolidating the apex region immediately N-terminal to the RWFD anchor. In high-scoring solutions for 9C2, 9C11, and 9C101, the CDRH3 tip features repeated engagement of residues 106–108 together with the heavy-chain rim R110 and a stabilizing VL ring (Y33/Y93/R95/S98), producing dense, reproducible contacts at the paratope-epitope ridge.

Notably, positions 102 and 105 in CDRH3 recur as interface nodes across multiple clonotypes, reinforcing their role as fine-tuning levers for geometry and local chemistry at the apex. In 9B65 and 9C154, residue 102 is directly captured in ClusPro contacts (Y102 and S102, respectively), and it also features in 9C11 (T102), placing this position at the heart of the tip-ridge clasp across phylogenetically distinct branches. In parallel, residue 105 appears in several solutions (e.g., 9A91: Y105; 9C11: R105; 9C101: T105), situating it just upstream of the apex where subtle side-chain differences can modulate reach, polarity, and packing without disrupting the anchor that forms further downstream. Together, the repeated capture of 102 and 105 across diverse binders underscores their importance for shaping a productive tip conformation and contact density (Fig. S5).

On the antigen side, late-branch antibodies converge on a similar receptor-binding motif (RBM) corridor centered around the 346–357 ridge and the 483–505 band-while also sampling positions such as K417 and adjacent residues. High-scoring poses for 9C2, 9C11, 9C101, and 9C30 repeatedly target these segments, aligning the consolidated CDRH3 apex against the RBM ridge to maximize hydrogen-bonding and π/salt-bridge opportunities. This epitope reuse offers a mechanistic basis for the concordance between docking energies and neutralization tiers: as the paratope narrows and hardens at the apex, it locks more consistently into the same high-value RBM corridor (Fig. S4 & S5). In sum, ClusPro analysis supports a model in which progression from 9A91 to late-branch members reflects paratope focusing at the CDRH3 apex, accompanied by recurrent involvement of positions 102 and 105 and stabilization by R110 and a VL rim. This structural consolidation correlates with the observed potency gradient across the lineage, explaining how late-stage clones achieve both deeper docking energetics and more stereotyped receptor-binding motif (RBM) engagement.

### 3.5 Molecular dynamics simulation for stability check

Molecular dynamics simulations were performed to evaluate the stability, flexibility, and interaction persistence of antibody-RBD complexes across the 9C lineage. To bracket the evolutionary spectrum, representative members were selected across the maturation spectrum, comprising the early germline-proximal clone 9A91, the intermediate-affinity binder 9B65, and the late, high-potency clone 9C111. To visualize the molecular basis of antigen engagement across the 9C lineage, representative antibody-RBD complexes were modeled for 9A91 (germline-proximal), 9B65 (intermediate), and 9C111 (late high-potency). In each case, the CDRH3 loop (highlighted in red) inserts into a conserved pocket of the RBD surface, making extensive contacts that anchor the interface. Despite their shared binding mode, subtle differences in loop geometry and burial depth were evident: 9A91 adopted a more extended configuration, 9B65 showed partial penetration with reduced complementarity, whereas 9C111 displayed a compact, deeply seated loop conformation (Fig. S3). Backbone Cα RMSD traces reflected this maturation pathway: 9A91 underwent broad fluctuations (∼0.5–2.2 nm) before stabilizing near 1.5 nm, while 9B65 plateaued at ∼1.8 nm, indicating greater instability. In contrast, 9C111 remained highly constrained (0.4–1.0 nm) throughout, consistent with a compact, stable apex (Fig. 2A). Residue-level RMSF maps reinforced this trend, with 9A91 and 9B65 showing pronounced mobility in CDRH3 (up to 1.7 nm), whereas 9C111 maintained minimal fluctuation (<0.6 nm) across the binding interface (Fig. 2B). The radius of gyration (Rg) and hydrogen bond (HB) counts supported these differences: 9C111 preserved a compact structure (∼2.5 nm) and stable HB network (45–55 bonds), while 9A91/9B65 remained more extended (2.9–3.0 nm) with fluctuating contacts (30–45 bonds) (Fig. 2C-D). Together, these data highlight how lineage maturation stabilizes CDRH3 geometry, yielding more consistent and durable antigen engagement. Consistent with these structural metrics, energy decomposition profiles revealed stable potential energies on the order of 10^6^ J/mol for all three complexes (3.5 × 10^6^ for 9A91, 6.0 × 10^6^ for 9B65, and 3.8 × 10^6 J/mol for 9C111), with only small-amplitude kinetic and conserved total energy fluctuations around zero, indicating well-equilibrated trajectories (Fig. 2E). Together, these data highlight how lineage maturation stabilizes CDRH3 geometry, yielding more consistent and durable antigen engagement.

**Fig. 2:**
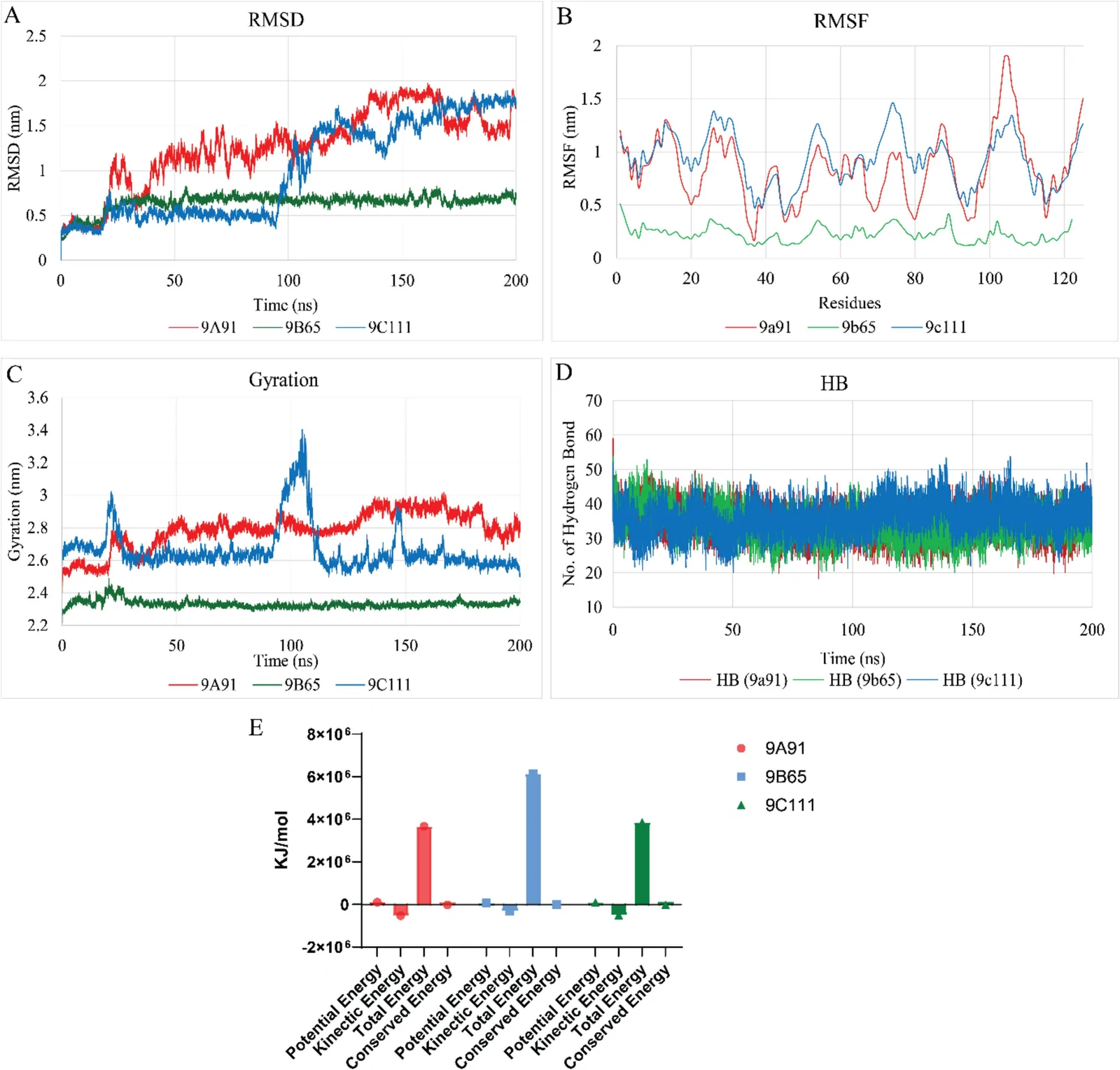
Structural Dynamics of Antibody-RBD Complexes. This figure presents the structural dynamics of three antibody-RBD complexes (9A91 in red, 9B65 in blue, and 9C111 in green) during molecular dynamics simulations. Subfigure A shows the RMSD (Root Mean Square Deviation) of the complexes over time, reflecting structural deviations. Subfigure B illustrates the RMSF (Root Mean Square Fluctuation), highlighting residue flexibility. Subfigure C displays the Radius of Gyration (Rg), indicating the compactness of each complex. Subfigure D presents the Hydrogen Bonding (HB) analysis, showing the number of hydrogen bonds formed between each antibody and the RBD. Subfigure E summarizes the average potential, kinetic, and conserved total energies for each antibody-RBD complex (potential energy 3.5 × 10 (Tas et al., 2016), 6.0 × 10 (Tas et al., 2016), and 3.8 × 10^6^ J/mol for 9A91, 9B65, and 9C111, respectively), confirming that all simulations remained energetically stable and directly comparable. GROMACS Version 2021.4 was used to generate all plots. The energy graphs generated in GraphPad prism 10.4.1. (For interpretation of the references to colour in this figure legend, the reader is referred to the web version of this article.)

A second MD set examined mature lineage members with varying apex conformations (9C111, 9C30, 9C101, 9C2). Structural models of mature antibodies illustrate distinct CDRH3 conformations across the lineage. (Fig. S4A) 9C30 adopts a partially extended loop (Fig. S4B), 9C101 shows an intermediate conformation with moderate burial, and (Fig. S4C) 9C2 displays a more open, flexible loop orientation. These differences mirror the dynamic stability hierarchy observed in MD simulations (Fig. 3). Here, RMSD and Rg analyses revealed that 9C111 remained the most stable (RMSD ∼1.5 nm; Rg ∼2.5 nm), while 9C30 and 9C101 displayed moderate excursions (∼2.0–2.5 nm; Rg ∼2.8–2.9 nm), and 9C2 exhibited the largest fluctuations (>2.5 nm; Rg ∼3.0 nm) (Fig. 3A-C). Importantly, RMSF profiles localized motion in 9C30 and 9C101 to peripheral loops, while the CDRH3 apex itself remained restrained, indicating that even flexible frameworks can preserve a stable paratope. HB analysis confirmed this hierarchy: 9C111 sustained the most stable interface (45–55 bonds), 9C30/9C101 showed intermediate stability (30–40 bonds), and 9C2 displayed fewer persistent interactions (20–30 bonds) (Fig. 3D). Energy decomposition further supported these trends (Fig. 3E): all complexes exhibited conserved total energies centered near zero, while potential energy values separated according to stability 9C111 and 9C101 showing the most favorable profiles (4–5 × 10^6^ J/mol), 9C30 displaying moderate values (3 × 10^6^ J/mol), and 9C2 maintaining the highest, least favorable potential energy (5–6 × 10^6^ J/mol). Kinetic energy distributions remained consistent across complexes, confirming comparable thermodynamic conditions during simulation.

**Fig. 3:**
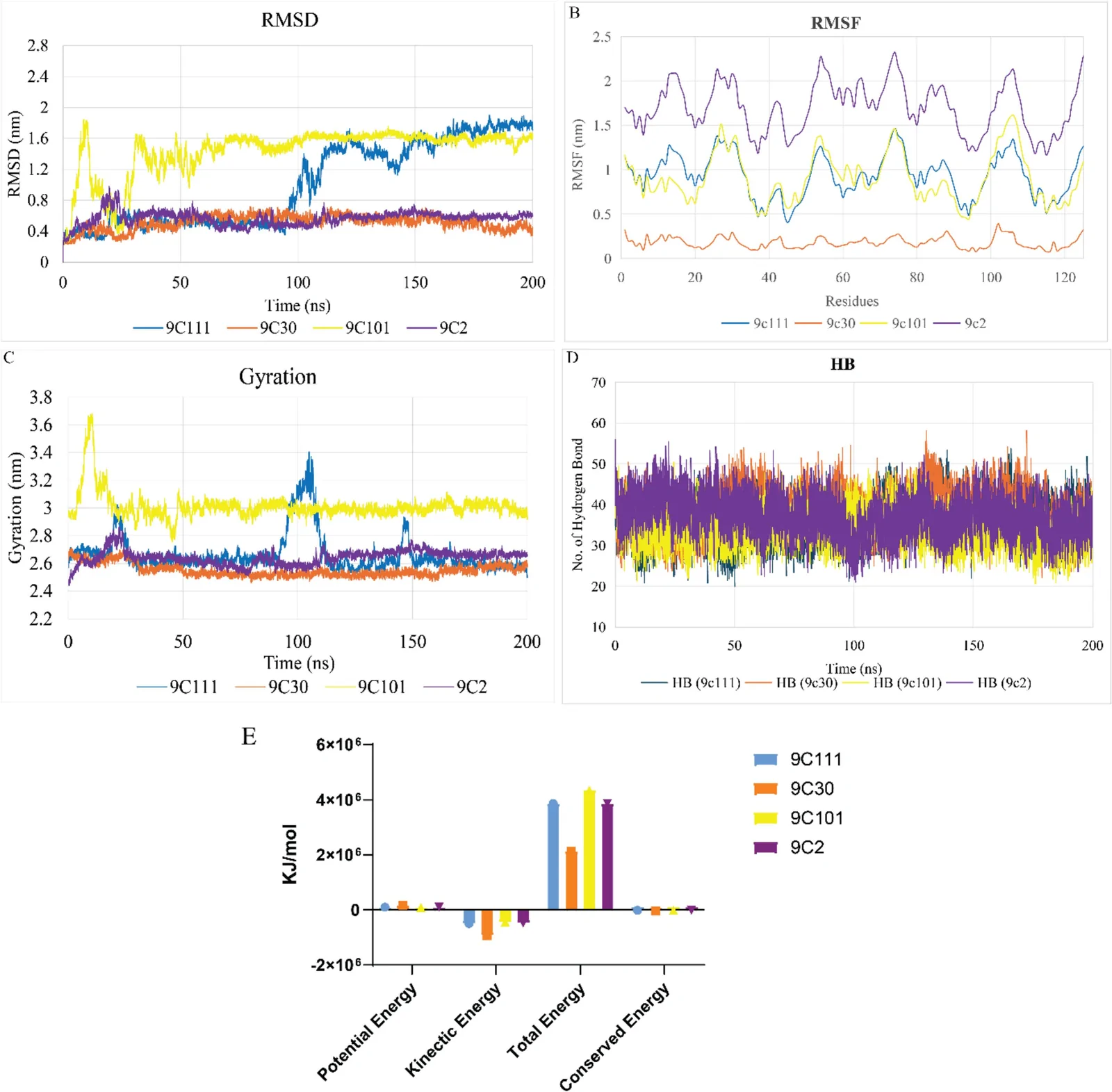
Structural Dynamics of Antibody-RBD Complexes. This figure presents the structural dynamics of four antibody-RBD complexes (9C111 in blue, 9C30 in green, 9C101 in yellow, and 9C2 in purple) during molecular dynamics simulations. Subfigure A shows the RMSD (Root Mean Square Deviation) of the complexes over time, reflecting structural deviations. Subfigure B illustrates the RMSF (Root Mean Square Fluctuation), highlighting residue flexibility. Subfigure C displays the Radius of Gyration (Rg), indicating the compactness of each complex. Subfigure D presents the Hydrogen Bonding (HB) analysis, showing the number of hydrogen bonds formed between each antibody and the RBD. Subfigure E presents average potential, kinetic, total, and conserved energies, showing that 9C111 and 9C101 maintain the most favorable potential energy (4–5 × 10^6^ J/mol), 9C30 occupies an intermediate position (3 × 10^6^ J/mol), and 9C2 displays the least favorable profile (5–6 × 10^6^ J/mol), while all complexes exhibit stable total and conserved energies. GROMACS Version 2021.4 was used to generate all plots. The energy graphs generated in GraphPad prism 10.4.1. (For interpretation of the references to colour in this figure legend, the reader is referred to the web version of this article.)

Finally, comparisons of 9C154 and 9C11 revealed two alternative late-branch strategies (Fig. 4). Structural comparison of late-branch members revealed two distinct stability strategies. 9C154 adopts a rigid, compact CDRH3 conformation with limited flexibility, whereas 9C11 maintains a more flexible apex but compensates with a dense hydrogen-bonding network. These alternative solutions highlight convergent modes of maintaining effective antigen engagement. 9C154 adopted a rigid, compact conformation (RMSD ∼1.0 nm; Rg ∼2.7 nm) (Fig. A &C) with moderate HB stability (35–45 bonds) (Fig. 4D), while 9C11 was more flexible (RMSD 1.0–1.5 nm; Rg ∼2.8 nm) but maintained a strong HB network (40–50 bonds). Energy decomposition further distinguished these two late-branch strategies (Fig. 4E). 9C154 displayed a moderately favorable potential energy (−5 × 10^5^ J/mol) with highly stable total energy (3.5 × 10^6^ J/mol), consistent with its rigid, compact architecture. In contrast, 9C11 exhibited slightly less favorable potential energy (−3 × 10^5^ J/mol) but maintained an even more stable total energy (3.8 × 10^6^ J/mol), aligning with its flexible yet strongly interacting paratope. Both complexes showed tightly clustered conserved energies near zero, confirming overall energetic stability during simulation. RMSF mapping suggested that 9C154 achieved stability by minimizing apex mobility, whereas 9C11 balanced flexibility with robust interaction density, reflecting different yet effective modes of stabilization (Fig. 4).

**Fig. 4:**
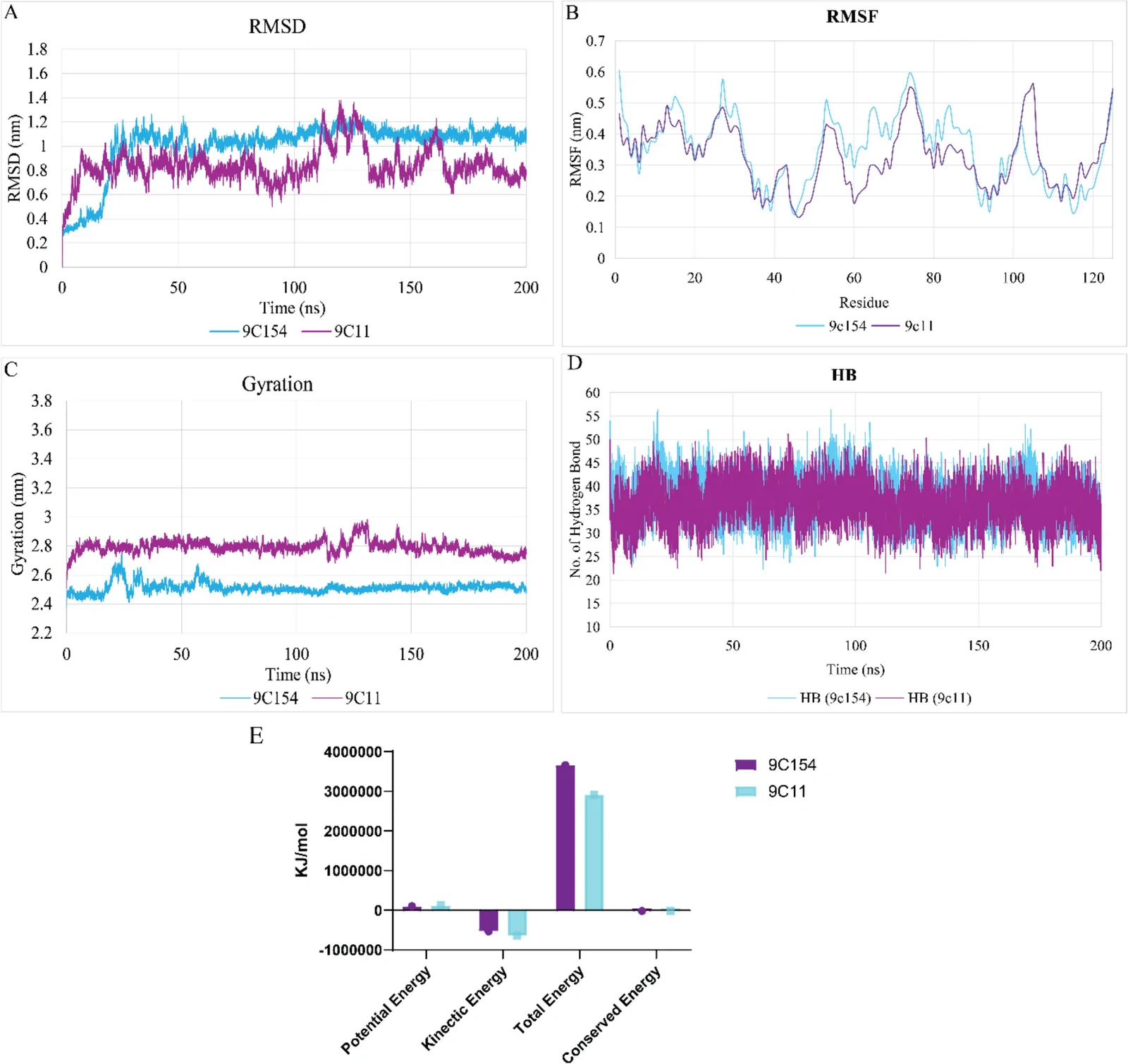
Structural Dynamics of Antibody-RBD Complexes. This figure presents the structural dynamics of the 9C154 (blue) and 9C11 (purple) antibody-RBD complexes during molecular dynamics simulations. Subfigure A shows the RMSD (Root Mean Square Deviation) of the complexes over time, reflecting the structural stability and deviations during the simulation. Subfigure B illustrates the RMSF (Root Mean Square Fluctuation), highlighting residue flexibility across the antibodies. Subfigure C displays the Radius of Gyration (Rg), indicating the compactness of each antibody-RBD complex during the simulation. Subfigure D presents the Hydrogen Bonding (HB) analysis, showing the number of stable hydrogen bonds formed between each antibody and the RBD throughout the simulation. Subfigure E summarizes potential, kinetic, total, and conserved energy profiles, showing that 9C154 maintains a more favorable potential energy (−5 × 10^5^ J/mol) and stable total energy (3.5 × 10^6^ J/mol), while 9C11 exhibits slightly higher potential energy (−3 × 10^5^ J/mol) but comparable overall energetic stability (3.8 × 10^6^ J/mol). GROMACS Version 2021.4 was used to generate all plots. The energy graphs generated in GraphPad prism 10.4.1. (For interpretation of the references to colour in this figure legend, the reader is referred to the web version of this article.)

In sum, MD simulations confirmed that maturation within the 9C lineage drives progressive stabilization of the CDRH3 apex. Stable high-potency clones (9C111, 9C11, 9C2) combine low RMSD, compact Rg, restrained apex mobility, and persistent HB networks, while early members remain flexible and energetically less stable. This dynamic trajectory complements docking and sequence analyses, reinforcing the central role of CDRH3 consolidation in functional antibody maturation.

### 3.6 Mutation antibodies interactions

The engineered series begins from 9A91 (WT), a germline-proximal precursor whose CDRH3 apex lacks the SYT cap immediately N-terminal to the invariant RWFD anchor. Starting from the germline-proximal 9A91, rigid-body docking revealed a weak interface (HDOCK -272.87; ClusPro −251.70), consistent with its unconsolidated CDRH3 apex. Installing the SYT cap (Mu9A91) at VH106–108 produced the most consistent improvement across engines (HDOCK -285.66; ClusPro −271.40), transforming the diffuse tip into a compact, polar/aromatic crest. Substitutions at flanking positions acted as modulators: Y105R alone partially eroded SYT gains, but in combination with G102A (4Mu9A91) yielded a cooperative boost, with HDOCK improving to −309.33. Across variants, docking confirmed that SYT installation was the dominant stabilizing edit, while positions 102 and 105 provided context-dependent fine-tuning (Table 3, Table S5).

**Table 3:** Molecular Docking Study and Interaction Analysis.

Antibody Name	Docking Score ClusPro	Antibody regions ClusPro	Antigen Regions ClusPro
9A91	−251.7	Heavy chain (A): GLU50, ASN52, SER56, THR57, SER58, MET64, ARG101, GLU104, TYR105, GLN106, LEU108, ARG110 Light Chain (B): TYR33, TYR93, ARG95	ARG346, ALA348, TYR351, ALA352, ASN354, ARG355, TYR396, PHE463, GLU464, ARG465, ILE467, SER468, THR469, GLU470, GLU515
MU9A91	−271.4	Heavy Chain (A): TYR33, SER56, GLU104, TYR105, SER106, THR108, ARG110 Light Chain (B): ARG95, SER98	THR345, ARG346, TYR351, ALA352, LYS443, GLY446, TYR448, LEU451, PHE489, GLN492, SER493
2MU9A91	−259.9	Heavy chain (A): TYR33, ASN52, SER56, THR57, ARG101, GLU104, ARG105, SER106, THR108, ARG110 Light Chain (B): TYR33, ARG95	LYS378, CYS379, VAL382, SER383, ARG408, PRO412, GLY413, GLN414, THR415, GLY416, ASP420, ASP427, ASP428, ASN459
3MU9A91	−273.0	Heavy chain (A): TYR33, SER56, GLU104, TYR105, SER106, THR108, ARG110 Light Chain (B): ARG95, SER98	LYS378, TYR380, SER383, PRO384, GLY413, THR415, ASP427, ASP428, PRO462
4MU9A91	−270.4	Heavy chain (A): TYR33, GLU50, ALA102, GLU104, ARG105, SER106, TYR107, THR108, ARG110 Light Chain (B): GLY32, TYR33, 34, ARG95, SER98	LYS417, ASP420, TYR421, LYS457, SER458, ASN459, GLU470, TYR472, GLN473, GLY475, SER476, PRO478, ASN486

Structural models of engineered 9A91 variants illustrate progressive stabilization of the CDRH3 apex. (Fig. S6A) Mu9A91 shows SYT installation forming a compact crest, (Fig. S6B) 2Mu9A91 and (Fig. S6C) 3Mu9A91 display incremental refinements, and (Fig. S6D) 4Mu9A91 combines SYT with flanking edits to achieve the most consolidated loop geometry. These conformational improvements align with docking predictions of enhanced binding.

Molecular dynamics simulations were conducted to evaluate how stepwise mutations modulate the conformational stability of the 9A91 antibody. Across 200 ns, wild-type 9A91 shows the broadest rise, increasing from ∼0.4 nm at launch to ∼1.6–1.8 nm at late times (100–200 ns), consistent with a flexible but gradually consolidating apex. Installing the SYT motif (Mu9A91) narrows the distribution overall (typically ∼0.9–1.4 nm after equilibration) but features a transient spike near ∼50 ns that reaches ∼2.0 nm. The double and triple edits (2Mu9A91, 3Mu9A91) further suppress large excursions, with traces largely constrained to ∼1.0–1.4 nm. The cumulative construct (4Mu9A91) remains in a similar window (∼1.0–1.5 nm) without prolonged high-amplitude events, indicating engineered variants collectively reduce sustained drift relative to the wild type while eliminating most outlier excursions (Fig. 5A). All constructs occupy a relatively tight Rg band, indicating no gross expansion or collapse of the antibody scaffold. Wild-type 9A91 fluctuates around ∼2.4–2.6 nm. Mu9A91 and 2Mu9A91 exhibit slightly higher and more variable envelopes early (∼2.6–2.9 nm), then converge toward ∼2.6–2.7 nm. 3Mu9A91 behaves similarly (∼2.6–2.8 nm). The cumulative variant (4MU9A91) remains in the compact range (∼2.5–2.7 nm) with modest drift. Taken together, Rg traces suggest that stepwise edits retain a compact molecular envelope; any transient widening introduced by intermediate variants is largely damped as edits accumulate (Fig. 5B). SASA differentiates the series more clearly than Rg. Wild-type 9A91 exhibits the lowest surface exposure (∼230–236 nm^2^). SYT installation increases SASA to ∼236–242 nm^2^ (Mu9A91), and this moderately elevated exposure persists for 2Mu9A91 and 3Mu9A91 (∼236–241 nm^2^). The cumulative 4Mu9A91 shows a similar or slightly higher band (∼236–242 nm^2^). Thus, while the global shape remains compact, engineered variants expose somewhat more solvent-accessible area, consistent with local loop re-packing that reorients polar side chains and enlarges the accessible crest without destabilizing the fold (Fig. 5C). Hydrogen-bond counts track the expected stability improvements. Wild-type 9A91 maintains a modest network (∼20–35 bonds). Mu9A91 increases this to ∼30–45 bonds. 2Mu9A91 and 3Mu9A91 sustain ∼35–50 bonds, reflecting enhanced but still dynamic interactions. The cumulative variant (4Mu9A91) supports the densest and most persistent network, ∼45–60 bonds throughout the trajectory, representing roughly a 50–60% increase over wild type and aligning with a more consolidated antigen-binding interface (Fig. 5D).

**Fig. 5:**
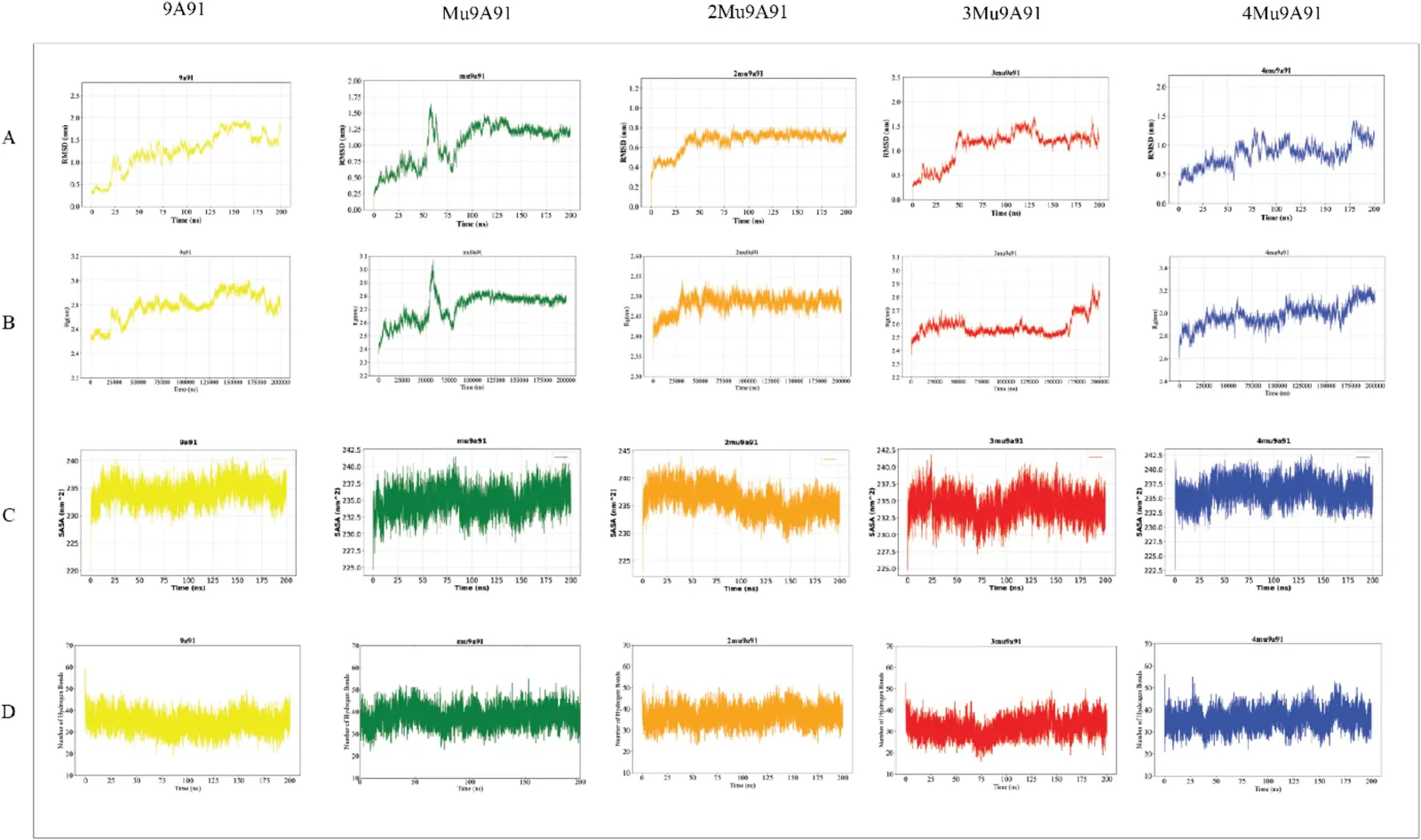
Molecular dynamics simulations of engineered 9A91 variants. (A) RMSD traces show that wild-type 9A91 exhibits broad fluctuations (∼0.5–2.0 nm) before stabilizing near 1.6–1.8 nm, while engineered variants progressively reduce drift, with 4Mu9A91 remaining below ∼1.5 nm after equilibration. (B) Radius of gyration (Rg) indicates all constructs retain compactness, with wild type at ∼2.4–2.6 nm and engineered variants converging near ∼2.5–2.7 nm. (C) Solvent-accessible surface area (SASA) highlights increased exposure in engineered variants (∼236–242 nm^2^) compared to wild type (∼230–236 nm^2^), consistent with loop remodeling. (D) Hydrogen bond analysis reveals a stepwise increase in interfacial stability, from ∼20–35 bonds in wild type to ∼45–60 in 4Mu9A91, reflecting the most persistent network of stabilizing interactions. GROMACS Version 2021.4 was used to generate all plots.

Energetic profiling supported these structural observations Across the 9A91 mutant panel, averaged energy decomposition revealed that all systems remained globally well behaved, but with clear mutation-dependent shifts in energetic favorability (Fig. 6). The wild-type 9A91 backbone showed moderately favorable potential energy (≈ −5.3 × 10^5^ J/mol) and balanced dynamics, with kinetic energy around 5.0 × 10^5^ J/mol and a total energy of ≈ 3.6 × 10^6^ J/mol. This profile reflects a relatively stable but only modestly optimized germline-proximal precursor (Fig. 6A). Installation of the SYT motif (Mu9A91) modestly improved this baseline. Mu9A91 exhibited a more favorable potential energy of ≈ −6.0 × 10^5^ J/mol while maintaining similar kinetic energy (∼4.8 × 10^5^ J/mol) and a total energy of ≈ 3.7 × 10^6^ J/mol (Fig. 6B). Addition of a second mutation (2Mu9A91) further deepened the potential energy well (≈ −7.0 × 10^5^ J/mol) and slightly raised the total energy to ≈ 4.3 × 10^6^ J/mol, indicating additional stabilization of internal contacts without compromising overall thermodynamic balance. Subsequent edits continued this trend of energetic refinement (Fig. 6C). The 3Mu9A91 variant reached a potential energy of ≈ −8.0 × 10^5^ J/mol with kinetic energy comparable to the other constructs (4.6 × 10^5^ J/mol) and a total energy again near 3.6 × 10^6^ J/mol, suggesting that added substitutions further optimize packing and electrostatics while preserving global dynamics (Fig. 6D). The cumulative 4Mu9A91 mutant achieved the most favorable energetic signature in the series. Its potential energy dropped to −9.0 × 10^5^ J/mol, and it uniquely displayed the lowest total energy (2.8 × 10^6^ J/mol), pointing to cooperative stabilization when all four edits are combined (Fig. 6E).

**Fig. 6:**
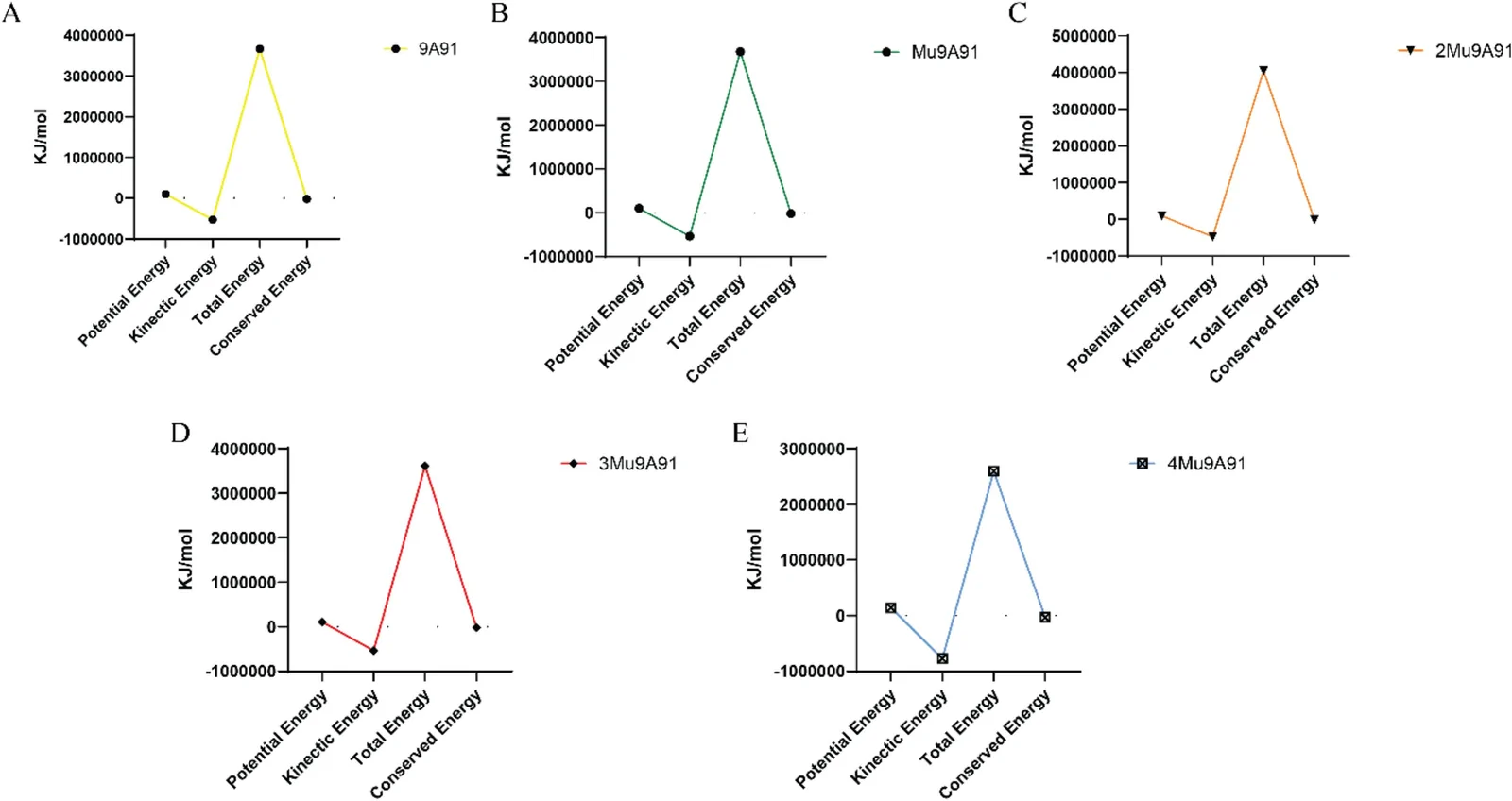
Comparative energy decomposition of 9A91 and engineered variants. (A-E) Averaged energy components (potential, kinetic, total, and conserved) for wild-type 9A91 and the Mu9A91, 2Mu9A91, 3Mu9A91, and 4Mu9A91 mutants. Each panel displays the mean energy values extracted from 200-ns MD simulations. Potential energy differences highlight mutation-dependent stabilization, while kinetic energy remains tightly clustered across variants. Total energy profiles reflect combined effects of kinetic and potential contributions, and conserved energy values remain near zero, confirming numerical stability and proper integration behavior during simulation. The energy graphs generated in GraphPad prism 10.4.1.

Molecular dynamics and energy analyses converge on the same principle: apex remodeling through SYT installation is the primary stabilizing driver, while positions 102 and 105 act as fine-tuning levers. The cumulative mutant (4MU9A91) achieves the greatest structural stability and energetic favorability, demonstrating how targeted edits can transform a weak germline-proximal precursor into a robust neutralizing framework.

### 3.7 SDS-PAGE assessment of purified antibodies

SDS-PAGE analysis was performed to assess the integrity of the purified parental and engineered antibodies from the 9C lineage. Under reducing conditions, the antibody preparations displayed two predominant bands corresponding to the immunoglobulin heavy and light chains, migrating at approximately 50 kDa and 25 kDa, respectively (Fig. S9). Comparable banding patterns were observed for 9A91 and its engineered derivatives (Mu9A91, 2Mu9A91, 3Mu9A91, and 4Mu9A91), as well as for the 9C-lineage antibodies and their corresponding mutants, including 9C2, Mu9C111, 2Mu9C111, 3Mu9C111, Mu9C101, 2Mu9C101, 3Mu9C101, 4Mu9C101, Mu9C11, Mu9C154, 2Mu9C154, A. Mu9C2, and I. Mu9C2. The consistent detection of both heavy- and light-chain components across the preparations confirmed successful recombinant expression and recovery of the antibody constructs and supported their suitability for subsequent binding and functional analyses.

### 3.8 Validation of mutations and evolution

Mutational variants of the germline-proximal antibody 9A91 were produced to test the contribution of CDRH3 edits on binding. ELISA absorbance (OD₄₅₀) confirmed that introduction of the SYT motif at positions 106–108 significantly enhanced binding over WT (Mu9A91: ∼27% stronger) with IC_50_ = 5.52 μg/mL (Fig. 7B)., whereas Y105R alone (2Mu9A91) retained activity comparable to wild type. G102A substitution (3Mu9A91) modestly increased binding (∼7%), while the combined G102A + Y105R edit (4Mu9A91) yielded the largest gain, improving binding by ∼64% with improved IC_50_ = 0.76 μg/mL (Fig. 7B). These results directly validate the structural predictions from docking: SYT installation is the major affinity driver, with positions 102 and 105 acting as modulators that can synergize to stabilize the apex (Fig. 7A).

**Fig. 7:**
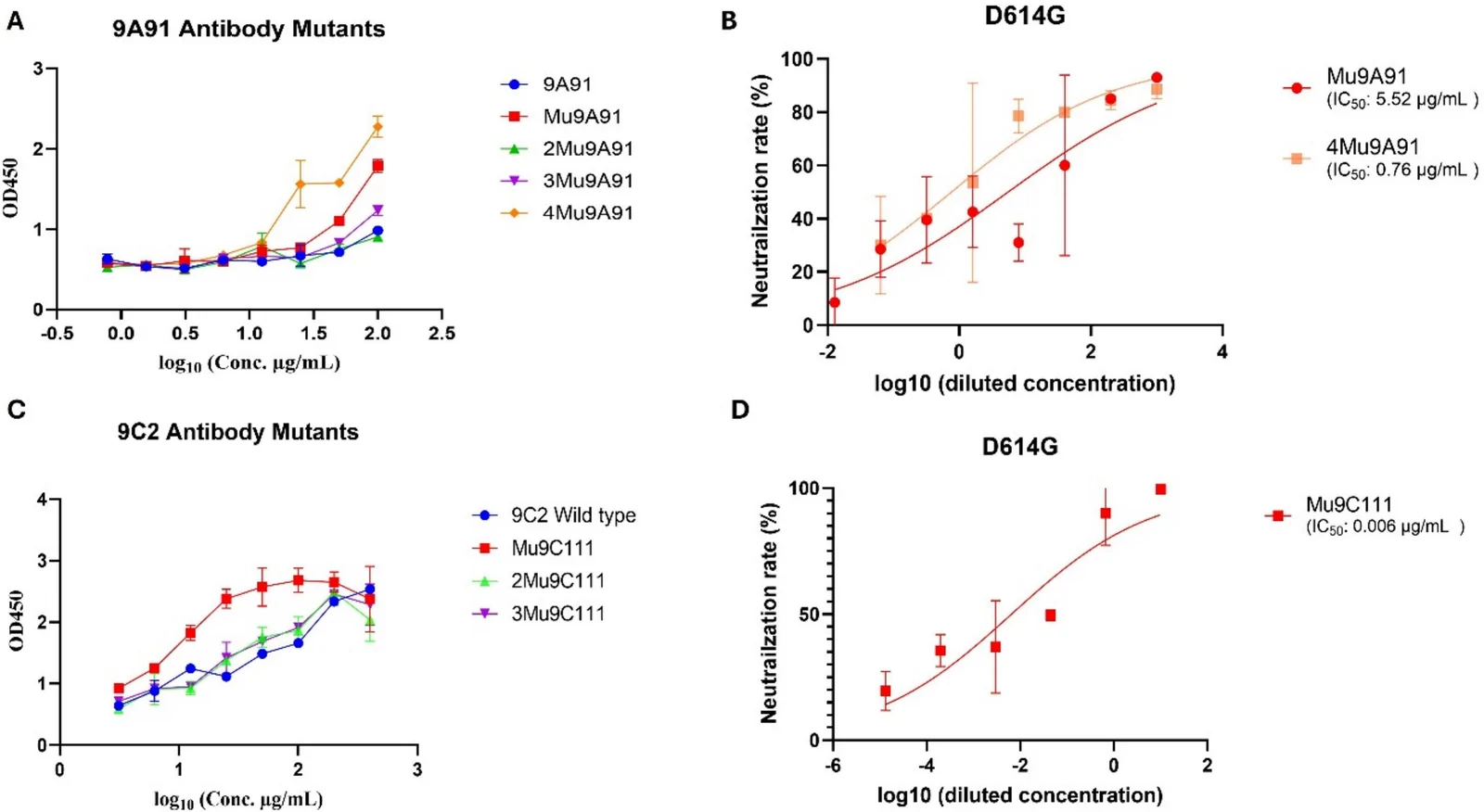
Functional validation of 9A91 mutants as well as 9C2 synonymous mutants by ELISA and Neutralization. (A) OD₄₅₀ absorbance curves of wild-type 9A91 (blue) and engineered variants Mu9A91 (red, SYT106–108), 2Mu9A91 (green, Y105R), 3Mu9A91 (purple, G102A), and 4Mu9A91 (orange, G102A + Y105R + SYT106–108) are shown across increasing antibody concentrations. The SYT-only variant (Mu9A91) exhibited ∼27% stronger binding relative to WT, while G102A alone (3Mu9A91) provided a modest ∼7% gain. The Y105R single mutant (2Mu9A91) showed binding comparable to WT. The combined triple edit (4Mu9A91) yielded the largest improvement (∼64% stronger binding). (B) The D614G pseudo-virus neutralization results of Mu9A91 (red) and 4Mu9A91 (orange). Data confirms that apex consolidation via SYT installation drives primary affinity gains and improvement in IC_50_, with flanking edits at positions 102 and 105 acting as modulators. Mean percent neutralization ± SD plotted in GraphPad prism 10.4.1 with 5-fold serial dilution. (C) OD₄₅₀ absorbance curves of wild-type 9C2 (blue) and engineered variants Mu9C111 (red), 2Mu9C111 (green), 3Mu9C111 (purple) are shown across increasing antibody concentrations. The only variant (Mu9C111) exhibited ∼40% stronger binding relative to WT. (D) The D614G pseudo-virus neutralization results of Mu9C111 (red). Mu9C111 exhibited altered RBD-binding and neutralization activity relative to the corresponding parental antibody; however, because the nucleotide substitution does not alter the encoded amino acid, this difference was not interpreted as evidence of a direct change in the protein-level binding interface. Elisa data are presented as mean ± SD from two technical replicates (*n* = 2) and plotted on a log10 concentration scale. Mean percent neutralization ± SD plotted in GraphPad prism 10.4.1 with 5-fold serial dilution. (For interpretation of the references to colour in this figure legend, the reader is referred to the web version of this article.)

Comparative sequence alignment of late-branch antibodies revealed that 9C111 (IC₅₀ = 0.022 μg/mL) and 9C101 (IC₅₀ = 0.120 μg/mL) differ at only two sites: position 102 (Thr vs. Ile) and position 98 (Ser vs. Ala), respectively. To assess the contribution of these residues to antigen binding, both synonymous and non-synonymous variants were generated (Fig. 7C, Fig. S7A, Table S6). At position 102, threonine in 9C111 is encoded by multiple codons (ACT, ACC, ACA, ACG), whereas in 9C2 the wild-type residue is isoleucine (ATT). To test the impact of this substitution, we inverted the 9C2 background into the 9C111 variant by introducing the I102T mutation (ATT → ACT; I. Mu9C2). Similarly, at position 98, serine in 9C101 is encoded by alternative codons (TCC, TCA, TCT, TCG), while the 9C2 antibody contains alanine (GCC). To explore its effect, we generated the A98S mutation (GCC → TCC; A. Mu9C2) (Fig. S7B), thereby inverting 9C2 into the 9C101 variant. Together, these targeted substitutions allowed us to directly evaluate whether the individual differences at residues 98 and 102 account for the enhanced binding observed in 9C111 and 9C101 (Table S6). Variants of 9C111 carrying alternative synonymous codons at position 102 displayed differences in the ELISA response, ranging from an approximately 10% reduction to a 40% increase relative to the parental preparation, with Mu9C111 also showing enhanced neutralization activity (IC₅₀ = 0.006 μg/mL). Because these synonymous substitutions preserve the amino-acid sequence, these differences were not attributed to alteration of the antibody–RBD interaction interface. Rather, they may reflect differences associated with recombinant expression, protein processing, or preparation-dependent variability. Accordingly, these synonymous variants were excluded from the protein-level mechanistic interpretation of residues controlling antigen recognition. In contrast, the non-synonymous I102T and A98S substitutions directly altered the encoded residues and were therefore used to evaluate the structural contribution of positions 102 and 98 to antigen binding (Fig. 7D). These findings support positions 98 and especially102 as secondary determinants that fine-tune affinity within a SYT-capped context. To extend this analysis, sequence comparison of 9C11 (IC₅₀ = 0.020 μg/mL) and 9C154 (IC₅₀ = 0.160 μg/mL) highlighted substitutions at 102 and 103 (Fig. 4.10). Functional assays of reciprocal mutants demonstrated modest tuning effects: Mu9C154 showed near-identical binding to WT9C11 (∼2% difference), a synonymous variant at position 102 slightly increased binding (∼3%), whereas a double mutant (2Mu9C154) reduced binding by ∼15% (Fig. S6). These data confirm that residues 102 and 103 serve as context-dependent modulators of binding stability rather than primary determinants (Fig. 7 & S8).

Together, these results establish a two-tier mechanism of affinity maturation within the 9C lineage. Primary gains arise from the installation of the SYT cap at positions 106–108, which transforms the germline apex into a compact polar/aromatic motif. Secondary refinements occur at positions 98, 102, and 103, where subtle residue swaps modulate binding strength and reproducibility. This layered process-apex consolidation, followed by fine-tuning-captures the evolutionary trajectory from 9A91 to the high-potency late-branch members.

## 4 Discussion

This study explored the rational evolution of the 9C antibody lineage against SARS-CoV-2, to enhance neutralization potency through a combination of computational and experimental approaches. By integrating docking, molecular dynamics (MD) simulations, and targeted mutagenesis with wet-lab validation, we demonstrated how structure-guided design can emulate and accelerate affinity maturation (Beshnova et al., 2022). This dual in silico-in vitro framework provides both predictive insights and functional confirmation, strengthening the conclusions (Chen et al., 2021). The results revealed a clear trajectory of functional maturation across the lineage (Kushari et al., 2025). Early antibodies such as 9A91 displayed weak binding and poor neutralization (IC₅₀ ≈ 26.9 μg/mL), consistent with their open, flexible CDRH3 apex. In contrast, late-lineage members like 9C111 and 9C2 evolved compact, interaction-dense apices centered on the S106-Y107-T108 (“SYT cap”) motif immediately preceding the RWFD anchor (Holt et al., 2023). These structural changes translated into striking potency gains, with 9C2 reaching an IC₅₀ of ∼0.009 μg/mL, representing nearly a 3000-fold improvement over 9A91. Computational analyses corroborated these findings: docking predicted progressively stronger binding energies along the lineage, while MD simulations showed reduced RMSF values and increased hydrogen bond stability in the high-potency clones (Halder et al., 2023). Importantly, engineered variants of 9A91 recapitulated these trends. The introduction of the SYT cap (MU9A91) alone markedly improved docking scores, stability, binding and neutralization IC_50_ = 5.52 relative to wild-type 9A91 and cumulative mutations (4Mu9A91) produced a phenotype that closely mirrored natural late-lineage antibodies. Wet-lab ELISA and neutralization testing confirmed these computational predictions, with 4Mu9A91 showing a ∼ 64% increase in binding and IC_50_ = 0.76 relative to wild-type 9A91. The convergence of silico and experimental evidence strengthens the case that CDRH3 remodeling is the principal driver of improved neutralization.

These findings are consistent with recent studies of SARS-CoV-2 antibody maturation. Longitudinal analyses of convalescent patients have shown that somatic hypermutation yields antibodies with orders-of-magnitude higher potency and broader recognition of variants (Muecksch et al., 2021) (Muecksch et al., 2021). Structural studies similarly highlight how affinity maturation often introduces turn-stabilizing residues or hydrogen bond networks in CDR loops, preconfiguring the paratope for high-affinity binding. Comparable to our SYT-cap remodeling, Moriyama et al. (2023, *Nature*) (Moriyama et al., 2023) used computational mutagenesis to engineer antibodies capable of neutralizing Omicron subvariants by reinforcing conserved RBD contacts. Together, these studies and our results point to a convergent evolutionary solution: polarity and geometric stabilization of CDRH3 are recurrent strategies to achieve potent, resilient neutralization(Yoshida and Oda, 2025).

Looking ahead, our engineered construction provides an opportunity to extend this work into functional breadth studies. In vitro neutralization assays against circulating and emerging SARS-CoV-2 variants, particularly those carrying escape mutations such as F486V or K444T, will be critical to validate whether SYT-stabilized apices can resist immune evasion. Parallel biophysical assays should confirm that these mutations not only enhance binding but also preserve favorable expression and stability profiles necessary for therapeutic development. The ability to engineer germline-like precursors into potent neutralizers suggests a strategy for rapidly updating antibody therapeutics as viral evolution continues.

This study highlights the 9C lineage as a model for understanding and harnessing antibody maturation. While installation of the compact SYT apex motif was a central driver of ultrapotent neutralization, our data also underscores the indispensable contribution of the invariant RWFD anchor, the modulatory roles of residues 98, 102, and 105, and alternative stabilization strategies observed in late-branch members such as 9C154 and 9C11. Together, these features reveal that potency arises from a layered architecture: apex consolidation, conserved anchoring, and context-dependent fine-tuning. By recapitulating these principles in engineered 9A91 variants, we demonstrate a generalized framework for antibody optimization. This integrative approach highlights how rational design can accelerate natural evolutionary solutions and provides a blueprint for developing broadly neutralizing antibodies to counter current and future viral threats.

## 5 Conclusions

This study establishes the 9C lineage as a robust model for understanding the structural and evolutionary principles of antibody maturation against SARS-CoV-2. We demonstrate that ultrapotent neutralization emerges from a layered process: installation of the SYT motif consolidates the CDRH3 apex, the invariant RWFD anchor safeguards antigen recognition, and context-dependent substitutions at positions such as 98, 102, and 105 fine-tune stability and binding. Alternative stabilization strategies observed among late-branch clones further highlight the convergent solutions by which antibodies achieve high potency. Importantly, engineered variants of the germline-proximal 9A91 reproduced these maturation principles, confirming that rational design can accelerate natural evolutionary pathways. Together, these findings provide both mechanistic insight into vaccine-induced antibody responses and a blueprint for engineering broadly neutralizing therapeutics capable of countering current and future SARS-CoV-2 variants.

## CRediT authorship contribution statement

**Muhammad Waqas Nasir:** Writing – original draft, Visualization, Project administration, Formal analysis, Data curation, Conceptualization, Writing – review & editing. **Qiyun Liang:** Resources, Formal analysis. **Jing Wang:** Project administration, Formal analysis. **Yan Sun:** Project administration, Formal analysis. **Xiangyu Zhang:** Project administration, Formal analysis. **Rishen Liang:** Project administration, Formal analysis. **Jun He:** Project administration, Formal analysis. **Umer Rashid:** Writing – review & editing, Software, Project administration. **Jiabing Wu:** Writing – original draft. **Yong Gao:** Writing – review & editing, Visualization, Validation, Supervision, Project administration, Funding acquisition, Conceptualization.

## Funding resources

Anhui Provincial Natural Science Foundation Project “In Vivo Induction and Regulation of Anti-HIV Broad-spectrum Neutralizing Antibodies” (2208085MH260).

## Declaration of competing interest

The authors declare that they have no known competing financial interests or personal relationships that could have appeared to influence the work reported in this paper.

## Data Availability

Data will be made available on request.

## Appendix A Supplementary data

Supplementary Material: Additional supporting data for this article.
