# Supplementary material for: Evolutionary and structural pathways of antibody maturation reveal principles for engineering potent SARS-CoV-2 neutralizers
Source: J Struct Biol X. 2026 Sep 18;14:100161. doi: 10.1016/j.yjsbx.2026.100161 (PMC13636076; doi:10.1016/j.yjsbx.2026.100161)
Supplement: Supplementary file 1 [file mmc1.docx]

Supplementary information for

**Evolutionary and Structural Pathways of Antibody Maturation Reveal Principles for Engineering Potent SARS-CoV-2 Neutralizers**

Muhammad Waqas Nasir^1^, Qiyun Liang^1^, Jing Wang^1^, Yan Sun^2^, Xiangyu Zhang^1^, Rishen Liang^1^, Jun He^3,4^, Umer Rashid^5*^, Jiabing Wu^3*^, Yong Gao^1*^

^1^ The First Affiliated Hospital of USTC, Division of Life Sciences and Medicine, University of Science and Technology of China, Hefei, Anhui, 230001, China

^2^ Department of Chemistry, University of Science and Technology of China, Hefei, Anhui, 230001, China

^3^Anhui Provincial Center for Disease Control and Prevention, Hefei 230601, China

^4^ Hefei Infectious Disease Hospital, Hefei, 230001, China

^5^ Department of Chemistry, COMSATS University, Islamabad, Abbottabad Campus, 22060, Abbottabad, Pakistan

* Corresponding Author:

Umer Rashid, ([umerrashid@cuiatd.edu.pk](mailto:umerrashid@cuiatd.edu.pk)).

Jiabing Wu, ([wjb0386@126.com](mailto:wjb0386@126.com)).

Yong Gao, (ygao387@ustc.edu.cn).

**Figure S1. CDRH3 alignment, clonal lineage, and neutralization potency of IGHV4-34 antibodies from vaccinee #9.** At left Alignment of CDRH3 amino acid sequences from eight clonotypes. Conserved residues are shaded, with the SYTV motif highlighted upstream of the invariant RWFD anchor. Right: Lineage diagram showing clonal relationships. Parental clones 9A91 and 9B65 display weak neutralization (high IC₅₀), while successive diversification yielded intermediate (9C154, 9C30, 9C101) and high-potency (9C11, 9C111, 9C2) lineages. IC₅₀ values are annotated below each clone. Node numbers indicate amino acid substitutions. Engineered variants of 9A91 (Mu9A91-4Mu9A91) are shown at right. Color scale denotes neutralization potency, from high IC₅₀ to low IC₅₀, A to C.

**Figure S2. Structural models of the 9C lineage Fv (panel order: A 9A91, B 9B65, C 9C111, D 9C154, E 9C30, F 9C101, G 9C11, H 9C2). Cyan, heavy-chain framework; pink, light-chain framework; red, heavy-chain CDRH3.** Frameworks are conserved across clones, whereas CDRH3 undergoes a directional transformation from short and kinked (A-B) to elongated and turn-stabilized (C, G, H), converging on a compact cap immediately upstream of RWFD. This structural convergence parallels the sequence/SHM signatures and explains the superior neutralization of late clones.

**Figure S3. Antibody-RBD interaction interfaces across the 9C lineage.** Structural models of representative antibodies bound to the RBD are shown, highlighting CDRH3 (red) insertion into the antigen surface pocket. (A) 9A91 (germline-proximal) adopts an extended conformation with limited burial of CDRH3. (B) 9B65 (intermediate) shows partial loop insertion and reduced complementarity. (C) 9C111 (late, high potency) displays a compact, deeply seated loop conformation that maximizes contacts. Right panels show close-up surface views of the RBD pocket with CDRH3 highlighted.

**Figure S4. Antibody-RBD interaction interfaces of mature lineage members.** Structural models of late-branch antibodies bound to the RBD are shown, with CDRH3 loops highlighted in red. (A) 9C30 adopts a partially extended loop conformation, (B) 9C101 displays an intermediate insertion with moderate burial, and (C) 9C2 exhibits a more open, flexible orientation. Insects show close-up surface views of the RBD pocket, illustrating differences in CDRH3 engagement that parallel the stability hierarchy observed in molecular dynamics simulations.

**Figure S5. Alternative stabilization strategies among late-branch antibodies.** Structural models of RBD-bound antibodies highlight CDRH3 loops in red. (A) 9C154 adopts a rigid, compact apex conformation with minimal flexibility, while (B) 9C11 maintains a more flexible loop but compensates through a dense hydrogen-bonding network. Insects show close-up views of the RBD interface, illustrating distinct yet effective solutions for achieving stable antigen engagement.

**Figure S6. Structural representations of the mu9a91.** (A), 2mu9a91 (B), 3mu9a91 (C), and 4mu9a91 (D) complexes. Each complex is shown in cartoon representation with domains colored green, purple, and cyan, and the binding region highlighted in red. The right panels display zoomed-in surface views of the binding interface, emphasizing the red β-strand interactions within the binding pocket.

**Figure S7. OD450 absorbance results of 9C11, 9C154 synonymous Mutations and Non-Synonymous Mutations at position 102 and 103**. A show the non-synonymous Mutations mutant’s absorbance at position 102,103 by replacing the T, S with S, T and vice versa. B shows the synonymous Mutations mutants’ absorbance at position 102 by replacing the ACT with AGT. Mu9c154 Fold change: ~0.98× against 9c11WT, Difference: ~2% decrease in binding vs WT9c11, Mu9c154: ~1.03-fold (≈ +3% higher than WT), 2Mu9c154: ~0.85-fold (≈ 15% lower than WT9c154). Data are presented as mean ± SD from two technical replicates (n = 2) and plotted on a log10 concentration scale.

**Figure S8: OD450 absorbance results of synonymous Mutations of 9C2 at 98 position, and Non-Synonymous Mutations at positions 102 and 98**. A shows the synonymous Mutations mutants absorbance at position 98 by replacing the A with S. B shows the non-synonymous Mutations mutants absorbance by replacing the 9C2 amino acids S and T at 98 and 102 positions with amino acids of 9C111 I and 9C101 A. Data are presented as mean ± SD from two technical replicates (n = 2) and plotted on a log10 concentration scale.

**Figure S9. SDS-PAGE characterization of purified parental and engineered antibodies from the 9C lineage.** (A) SDS-PAGE profiles of 9A91 and its engineered variants Mu9A91, 2Mu9A91, 3Mu9A91, and 4Mu9A91. (B) SDS-PAGE profiles of 9C2, Mu9C111, 2Mu9C111, and 3Mu9C111 (left), and Mu9C101, 2Mu9C101, 3Mu9C101, and 4Mu9C101 (right). (C) SDS-PAGE profiles of Mu9C11, Mu9C154, and 2Mu9C154 (left), and A.Mu9C2 and I.Mu9C2 (right). The antibody preparations exhibited the characteristic heavy- and light-chain bands, with a 25-180 kDa molecular-weight marker included for reference, supporting successful expression and purification of the recombinant antibody constructs.

**Table S1:** V(D)J gene usage and CDR3 features of IGHV4-34 clonotypes.

| **Clone** | **V gene**  **(ID %)** | **D gene**  **(ID %)** | **J gene**  **(ID %)** | **CDR3 length (AA)** | **CDR3 motif** |
| --- | --- | --- | --- | --- | --- |
| 9A91 | IGHV4-34*01 (93.9) | IGHD2-2*02 (100.0) | IGHJ5*02 (100.0) | 19 | ARGPRRRGEYQLLYRWFDP |
| 9B65 | IGHV4-34*01 (93.5) | IGHD2-2*03 (100.0) | IGHJ5*02 (100.0) | 15 | ARGYNGYSPGQRWFDP |
| 9C111 | IGHV4-34*01 (92.3) | IGHD2-2*02 (91.2) | IGHJ5*02 (100.0) | 18 | VGSADWITPTSYTVRWFDS |
| 9C11 | IGHV4-34*01 (93.5) | IGHD2-2*02 (85.2) | IGHJ5*02 (100.0) | 18 | ARGLYCTSVRCYTSRWFDP |
| 9C154 | IGHV4-34*01 (93.5) | IGHD2-2*02 (85.2) | IGHJ5*02 (100.0) | 18 | ARGLYCSTVRCYTSRWFDP |
| 9C30 | IGHV4-34*01 (93.8) | IGHD2-2*02 (84.0) | IGHJ5*01/02 (97.8) | 18 | ARSSHCASSSCSTVRWFDS |
| 9C101 | IGHV4-34*01 (93.2) | IGHD4-11/40 (100.0) | IGHJ5*01/02 (97.8) | 18 | VGAADWTTPTSYTVRWFDS |
| 9C2 | IGHV4-34*01 (93.1) | IGHD3-16*03 (100.0) | IGHJ5*01/02 (97.8) | 18 | VGSADWITPTSYTVRWFDS |

**Table S2: Optimization of tools for modelling on the basis of XG83 Fv heavy chain (Hc), light chain (Lc) models RMSD calculation after superposition with XG83 antibody crystallography model.**

| **Tools and Software** | **Fv LC, HC RMSD** | **Fv LC RMSD** | **Fv HC RMSD** | **CDRH3 RMSD** | **CDRH1, H2**  **RMSD** | **CDRL3 RMSD** | **CDRL1, L2**  **RMSD** |
| --- | --- | --- | --- | --- | --- | --- | --- |
| Abodybuilder2 Fv Modelling | 1.4 Å | 0.9 Å | 1.6 Å | 2.1 Å | 0.1 Å | 0.3 Å | 0.9 Å |
| MOE Fv  Modelling | 1.6 Å | 1.0 Å | 1.4 Å | 1.2 Å | 0.1 Å | 0.5 Å | 0.1 Å |
| I Tesser Fv  Modelling | 1.5 Å | 1.4 Å | 2.1 Å | 2.8 Å | 0.9 Å | 0.8 Å | 1.2 Å |
| Colab Fold AlphaFold 2 | 1.1 Å | 1.2 Å | 1.0 Å | 1.2 Å | 0.8 Å | 0.2 Å | 1.3 Å |
| Swiss Fv Modelling | 4.8 Å | 0.8 Å | 2.4 Å | 3.7 Å | 1.1 Å | 0.3 Å | 0.8 Å |

**Table S3:** Structural quality assessment before and after refinement

| **Antibody** | **Metric** | **Before** | **After** | **Benchmark** |
| --- | --- | --- | --- | --- |
| 9A91 | Clashscore (all atom) | 21.73 | **0.00** | < 0.30 |
|  | MolProbity score | 2.05 | **0.73** | < 2.0 |
|  | Ramachandran Z-score (mean ± SD) | 0.10 ± 0.49 | **1.10 ± 0.48** |  |
| 9B65 | Clashscore | 21.44 | **0.00** | < 0.30 |
|  | MolProbity score | 2.00 | **0.51** | < 2.0 |
|  | Ramachandran Z-score | 0.01 ± 0.49 | **1.04 ± 0.49** |  |
| 9C154 | Clashscore | 16.52 | **0.00** | < 0.30 |
|  | MolProbity score | 2.05 | **0.50** | < 2.0 |
|  | Ramachandran Z-score | −0.30 ± 0.50 | **0.71 ± 0.50** |  |
| 9C30 | Clashscore | 19.01 | **0.00** | < 0.30 |
|  | MolProbity score | 2.00 | **0.64** | < 2.0 |
|  | Ramachandran Z-score† | **0.34** ± 0.50 | **0.96 ± 0.48** |  |
| 9C101 | Clashscore | 13.75 | **0.00** | < 0.30 |
|  | MolProbity score | 1.87 | **0.50** | < 2.0 |
|  | Ramachandran Z-score | 0.13 ± 0.48 | **0.64 ± 0.48** |  |
| 9C111 | Clashscore | 14.86 | **0.00** | < 0.30 |
|  | MolProbity score | 1.94 | **0.50** | < 2.0 |
|  | Ramachandran Z-score | 0.05 ± 0.49 | **0.64 ± 0.48** |  |
| 9C11 | Clashscore | 13.75 | **0.00** | < 0.30 |
|  | MolProbity score | 1.87 | **0.50** | < 2.0 |
|  | Ramachandran Z-score | 0.34 ± 0.50 | **0.66 ± 0.50** |  |
| 9C2 | Clashscore | 14.31 | **0.00** | < 0.30 |
|  | MolProbity score | 1.93 | **0.50** | < 2.0 |
|  | Ramachandran Z-score | 0.03 ± 0.49 | **0.72 ± 0.48** |  |

**Table S4. Molecular Docking Study and Interaction analysis (HDock)**

| **Antibody Name** | **Model Number Hdock** | **Docking Score Hdock** | | **Antibody Regions Hdock** | **Antigen regions**  **Hdock** |
| --- | --- | --- | --- | --- | --- |
| 9A91 | 2 | -272.87 | Heavy chain (A): SER56, THR57, SER58, TYR59, ARG101, GLU104, GLN106, LEU107, LEU108, ARG110  Light Chain (B): SER26, ALA31, ASP93, ARG95, SER98 | | ARG346, ALA348, TYR351, ALA352, ASN354, ARG355, TYR396, PHE463, GLU464, ARG465, ILE467, SER468, THR469, GLU470, GLU515 |
| 9B65 | 2 | -285.35 | Heavy chain (A): THR17, TYR33, GLU50, SER54, GLY55, SER56, THR57, SER58, MET64, THR68, LYS81, SER83, GLY101, TYR102,  Light Chain (B): SER96, SER98 | | ARRG403, ARG408, LYS417, TYR421, TYR448, TYR452, PHE485, ASN486, TYR488, GLN492, SER493, GLY495, GLY501, VAL502, TYR504 |
| 9C111 | 2 | -305.97 | Heavy Chain (A): ASP31, ASN52, SER56, SER58, SER74, LYS76, TYR107, THR108  Light Chain (B): TYR93 | | GLY445, SER476, GLU483, PHE485, ASN486, TYR488, GLN492, GLN497 |
| 9C154 | 3 | -321.91 | Heavy Chain (A): PHE29, SER30, ASN52, HIS53, SER54, SER56, THR73, TYR100, SER102, VAL104, TYR107, ARG110 Light Chain (B): TYR93, ARG95, SER96, SER98 | | ARG346, TYR351, ALA325, ASN354, ARG355, ARG357, TYR396, LEU451, ARG465, ARG465, ILE467, SER468, GLY481, GLU483, PHE489 |
| 9C30 | 7 | -339.67 | Heavy Chain (A): HIS53, GLY55, THR57, TYR59, MET64, SER65, THR108, VAL109 Light Chain (B): TYR33, TYR93, ARG95, SER98 | | THR345, ARG355, LYS356, ARG357, TYR396, GLU464, ARG465, ASP466, ILE467 |
| 9C101 | 4 | -364.62 | Heavy Chain (A): GLU27, SER30, ASP31, TYR33, HIS53, SER56, SER58, ASP100, PRO104, THR105, SER106, THR108  Light Chain (B): TYR33, TYR93, ARG95, SER98 | | ARG346, SER349, TYR351, ARG355, ARG357, TYR396, ASN449, PHE463, GLU464, ARG465, ILE467, SER468, THR469, GLU470, GLY481 |
| 9C11 | 3 | -352.62 | Heavy Chain (A): TYR33, SER56, THR57, SER58, PRO61, TYR100, VAL104, ARG105, CYS106, THR108 Light Chain (B): ALA31, TYR33, ASP34, TYR93, ARG95, LEU97, SER98 | | LEU335, CYS336, PRO337, ARG346, ALA348, ASN354, ARG355, LYS356, ARG357, SER359, ASN360, ASN394, TYR396, ASN449, PHE463, GLU464, ARG465, GLU515, HIS518 |
| 9C2 | 2 | -366.53 | Heavy Chain (A): THR57, PRO61, MET64, THR68, LYS81, SER83, SER106, TYR107, THR108, ARG110 Light chain(B): TYR33, ASP94, ARG95, SER98 | | GLY404, ASP405, ARG408, LYS417, TYR452, LEU454, PHE455, PHE485, ASN486, TYR488, GLN492, ASN500, GLY501, VAL502, GLY503, GLY504, GLN505 |

**Table S5:** Molecular Docking Study of Mutations and Interaction analysis

| **Antibody Name** | **Model Number Hdock/ cluspro** | **Docking Score Hdock/ cluspro** | **Antibody Regions HDock** | **Antigen regions Hdock** |
| --- | --- | --- | --- | --- |
| 9a91 | 2 | -272.87 | Heavy chain (A): SER56, THR57, SER58, TYR59, ARG101, GLU104, GLN106, LEU107, LEU108, ARG110  Light Chain (B): SER26, ALA31, TYR93, ARG95, SER98 | ARG346, ALA348, TYR351, ALA352, ASN354, ARG355, TYR396, PHE463, GLU464, ARG465, ILE467, SER468, THR469, GLU470, GLU515 |
| MU9A91 | 3 | -271.4 | Heavy Chain (A): TYR33, GLU50, ARG101, TYR105, SER106, TYR107, THR108, ARG110  Light Chain (B): ALA31, VAL35, TYR93, ARG95, SER96, SER98 | THR345, ARG346, TYR351, ALA352, LYS443, GLY446, TYR448, LEU451, PHE489, GLN492, SER493 |
| 2MU9A91 | 3 | -259.9 | Heavy chain (A): TYR33, GLU50, SER54, SER58, ARG101, ARG105, SER106, TYR107, THR108, ARG110  Light Chain (B): ALA31, TYR33, ARG95, SER98 | ARG346, ALA348, SER349, TYR351, ALA352, LYS443, GLY445, GLY446, TYR448, ILE467, GLU483, PHE489, SER493, |
| 3MU9A91 | 2 | -273.0 | Heavy chain (A): TYR33, GLU50, ARG101, TYR105, SER106, TYR107, THR108, ARG110  Light Chain (B): ALA31, GLY32, TYR33, TYR93, ARG95, SER96, SER98 | THR345, ARG346, ALA348, SER349, TYR351, ALA352, LYS443, GLY446, TYR448, LEU451, PHE489, GLN492, SER493, |
| 4MU9A91 | 4 | -270.4 | Heavy chain (A): TYR33, ASN52, SER56, THR57, ARG105, TYR107, THR108, ARG110  Light Chain (B): GLY32, TYR33, GLY52, ASN54, ASN55 LYS68, SER69 | VAL444, GLY445, TYR448, ASN449. LEU451, GLU483, PHE485, TYR488, GLN492, SER493, GLN497, ASN500, TYR504 |

Table S6. Mutated antibodies names with nucleotide change and coding amino acid after Synonymous Mutations and Non-Synonymous Mutations at position 102 and 98.

| **Amino Acids** | **Nucleotides** | **Mutated Antibodies** |
| --- | --- | --- |
| Threonine (Thr) 102 position | ACT | 9C2 |
|  | ACC | Mu9C111 |
|  | ACA | 2Mu9C111 |
|  | ACG | 3Mu9C111 |
| Serine (Ser) 98 position | TCC | Mu9C101 |
|  | TCA | 2Mu9C101 |
|  | TCT | 3Mu9C101 |
|  | TCG | 4Mu9C101 |
| I102T position | ATT102ACT | I.Mu9c2 |
| A98S Position | GCC98TCC | A. Mu9c2 |
